# Metal‐Coordination Specificity and Structural Dynamics of *C. elegans* Metallothionein I: Insights From 3D Modeling and MD Simulations

**DOI:** 10.1002/prot.70054

**Published:** 2025-09-21

**Authors:** Nilvea Ramalho de Oliveira, Andrei Santos Siqueira, Paulo Sérgio Alves Bueno, Evonnildo Costa Gonçalves, Juliano Zanette

**Affiliations:** ^1^ Instituto de Ciências Biológicas Universidade Federal do Rio Grande Rio Grande Rio Grande do Sul Brazil; ^2^ Laboratório de Tecnologia Biomolecular, Instituto de Ciências Biológicas Universidade Federal do Pará Belém Pará Brazil; ^3^ Departamento de Bioquímica Universidade Estadual de Maringá Maringá Paraná Brazil

**Keywords:** AMBER, metalloprotein, molecular dynamic simulation, structural biology, tridimensional protein

## Abstract

Metallothioneins (MTLs) are small, cysteine‐rich proteins known for their ability to bind metal ions and exhibit flexible, disordered structures. The structural and functional characteristics of metallothionein I (MTL‐1) from *Caenorhabditis elegans were investigated*, focusing on its behavior in both metal free (MTL‐1 Apo) and metal‐bond states with Zn^2+^, Cd^2+^, Cu^2+^, Hg^2+^, and Pb^2+^ divalent metal ions. Using molecular dynamics simulations and 3D modeling via AlphaFold, we characterized the flexibility and stability of MTL. The MTL‐1 Apo form displayed high flexibility, aligning with its intrinsically disordered protein (IDP) nature, with 89.3% of its structure composed of coils, bends, and turns. Metal binding significantly enhanced the protein's stability, particularly with Zn^2+^, Cd^2+^, Cu^2+^, and Hg^2+^, reducing root mean square deviation (RMSD), root mean square fluctuation (RMSF), accessible surface area (SASA) and radius of gyration (*R*_g_) values, indicating structural compaction. Conversely, Pb^2+^ showed a weaker stabilizing effect, with a more dynamic and less stable structure. Structural analysis revealed that conserved cysteine residues coordinate the metal through strong thiolate interactions, with additional contributions from non‐cysteine residues, such as Glu and Lys. The study underscores the importance of incorporating intrinsically disordered protein models in MD simulations to provide deeper insights into how metallothionein's flexibility and stability vary in response to different metal ions, offering a structural perspective on their biological interactions and behavior under diverse environmental conditions. While thermodynamic aspects were not directly assessed, the results reveal consistent conformation trends across different metal coordination states.

## 1 Introduction

Metallothioneins (MTL) are ubiquitous and highly conserved proteins throughout biological systems of animals, higher plants, eukaryotic microorganisms, and many prokaryotes [1, 2, 3]. MTLs have been considered the first lines of defense against metal contamination among proteins related to detoxification. It is known that these proteins are involved in metal homeostasis, performing transport, storage, and detoxification [4]. They have a low molecular weight (6–7 kDA) and are rich in cysteines (> 25%) of comparatively higher sulfur content [5]. The binding to metals has been attributed to the presence and conserved distribution of these cysteine residues along the polypeptide chain.

In mammals, for example, four isoforms of metallothionein are present, MTL‐1 to MTL‐4 [3]. In the invertebrate model organism *Caenorhabditis elegans* (Nematoda, Rhabditidae), two forms can be found, MTL‐1 and MTL‐2, which contain 75 and 63 amino acid residues, respectively. *C. elegans* serves as a useful model for studying MTL function in response to metal exposure, with both isoforms playing critical roles in protection against metal toxicity [6]. Studies have indicated that *mtl‐1gene expression* is rapidly activated in response to metal exposure, showing a marked preference for Zn over Cd (CD spectra methods [7, 8]). In contrast, *mtl‐2 gene expression* appears to be primarily constitutive and localized in gut cells [9]. Consistent with these findings, only MTL‐1 appears to play a crucial role in protecting against depleted uranium (^238^U) contamination [10]. Therefore, MTL‐1 seems more important in physiological responses to metal exposure.

A wide range of metals can adversely affect organisms, particularly non‐essential metals, which have no biological function and are toxic to the organism in minimal amounts. However, even essential metals can become toxic if concentrations exceed safe limits [11]. Both kinds of metal ions can bind to MTL and affect their conformation, contributing to their biochemical activity in the biological system and consequently to its stability. Despite that, research has mainly focused on the effects of Zn^2+^ and Cd^2+^ on MTL isoforms in different species [12, 13, 14, 15, 16, 17]. From the structural perspective, albeit a small protein, metallothionein constitutes a highly complex system, with the same peptide with the ability to display different final 3D folds, which confers high flexibility [4]. In addition, they lack a well‐defined and stable tertiary and secondary structure in their apo forms (demetalated condition) aligned with characteristics of intrinsically disordered proteins (IDPs) [18]. This protein exhibits a flexible and highly dynamic conformation [19, 20, 21, 22]. The possible intrinsic disorder of MTs increases the complexity of evaluating and understanding their interactions with different metals.

Structural and functional characteristics of more stable proteins can be precisely determined using x‐ray crystallography and nuclear magnetic resonance spectroscopy [23]. However, these techniques have some limitations when applied to IDPs [9, 24]. In addition, these methods are expensive and require extensive effort and time. Recently, in silico studies accessing the three‐dimensional structure of proteins and molecular dynamics have generated significant progress in predicting their functionality [25, 26, 27]. In the case of metallothionein, investigations into the metal‐coordination sites have been carried out in order to understand their role in metabolism, as well as mechanisms involving toxicity [28, 29, 30]. Using computational models, we can gain insights into the dynamic behavior of MTLs and their roles in metal exposure, advancing our understanding of their biochemical functions and potential applications in managing metal toxicity.

In this context, the present study was aimed to perform a computational screening to explore how different divalent metal ions (Zn^2+^, Cd^2+^, Cu^2+^, Pb^2+^, and Hg^2+^) affect the conformation and molecular dynamics of isoform I of metallothionein from *C. elegans* (MTL‐1). To achieve this goal, we employed a structure‐based approach using an AlphaFold‐derived model of MTL‐1 and carried out all‐atom molecular dynamics simulations with each metal. The objective was to investigate general trends in in‐protein interaction propensity and to assess the potential for local stabilization near coordination residues, providing insights into how different divalent metal ions may influence the conformation and dynamics of MTL‐1. This theoretical screening enabled us to examine conformational flexibility, coordination sites, and dynamic features of the protein, expanding our understanding of MTL‐1 responses to different metals. The results from these simulations were compared with experimental data to support and complement our findings.

## 2 Materials and Methods

### 2.1 Molecular Modeling of MTL‐1 From *C. elegans*

The 3D structure of metallothionein I (MTL‐1) from *Caenorhabditis elegans* was obtained from the AlphaFold Protein Structure Database (AFDB) via the Foldseek plataform [31], using the accession P17511 AF‐P17511‐F1‐v4. This model was selected due to its higher structural quality based on validation metrics when compared with models generated using other approaches. To evaluate the quality of the selected model, we performed structural validation using several tools: pLDDT score [32], Ramachandran plot, QMEAN [33] and Molprobity score [34], ERRAT [35] and ProSA‐web [36]. Additionally, the model was visually inspected using PyMoL software (The PyMOL Molecular Graphics System, Version 2.5.2 Schrödinger LLC). The secondary structure was determined quantitatively using the DSSP program [37].

### 2.2 System Preparations for Molecular Dynamic Simulations

To investigate the conformation and structural stability of MTL‐1 coordinated with two different metals, we assembled six systems: one MTL‐1 Apo system without metal ions, which served as an exploratory control, allowing us to assess conformational flexibility in the absence of metals, and five Holo MTL‐1 systems, each loaded with seven ions of Zn^2+^, Cd^2+^, Cu^2+^, Pb^2+^, and Hg^2+^, respectively. All systems were derived from the AlphaFold‐predicted structure of MTL‐1, with a uniform protonation scheme applied across apo and holo models. Specifically, all cysteine residues were modeled in their deprotonated forms—thiolates (S^−^), consistent with the well‐established requirement for thiolate anions in divalent metal coordination [7, 38, 39]. Histidine residues were modeled in their neutral imidazole forms (HIE), which represent the standard tautomers available in the Amber force field.

The systems were neutralized by Na^+^ or Cl^−^ ions. The all‐atom molecular dynamic simulations (MD) were conducted in triplicate for each system using the Amber 23 software package [40]. Each MD simulation was run for 200 ns with a 2 femtoseconds (fs) integration step. Coordinates were written every 2 ps, yielding 100.000 frames per trajectory. For convenience in post‐processing, the trajectories were split into 10 ns segments, each containing 5.000 frames.

The results were analyzed in terms of (1) root mean square deviation (RMSD) of the backbone, which measures the overall changes in conformation from the reference structure; (2) root mean square fluctuation (RMSF), assessing the flexibility of individual residues over time to evaluate structural stability and dynamics of the system; (3) accessible surface area (SASA) determined by changes in the accessibility of protein to solvent using “surf” command in the cpptraj module; and finally (4) radius of gyration (*R*_g_), used as a measure of the compactness of a protein conformation. All saved frames were used for quantitative analyses. For structural visualization and illustrative purposes, 20 uniformly spaced snapshots were extracted from each trajectory.

For MD simulations, the MTL‐1 full metalated approach was adopted, where it can bind up to seven divalent metals distributed in two metal–cysteine clusters: four ions in the α‐protein domain and three ions in the β‐protein domain [8, 41, 42]. General amber force field (GAFF) [43] and the FF19SB [44] force fields for proteins were used. The systems were solvated in an octagonal box with 10 Å in each direction of the protein by optimal point charge (OPC) water molecules [45]. The divalent metal ions Zn^2+^, Cd^2+^, Cu^2+^, Pb^2+^, and Hg^2+^ were modeled as free ions using nonbonded approach with standard 12–6 Lennard–Jones parameters from frcmod.ionslm_126_opc file [46], loaded via leaprc.water.opc [47]. These parameters represent each metal as charged particle with empirically derived van der Walls properties, without explicit coordination or bonded interactions with the protein. Although this simplified representation does not account for coordination geometry, it is widely used for comparative studies of structural and dynamics effects of metal ions in biomolecular systems [48, 49].

Energy minimization was performed in five steps, using cycles of steepest descent and conjugate gradients. The heavy atoms were restrained by a harmonic potential of 1000 kcal mol^−1^ × Å^−2^ initially. In the last step, 5.000 cycles of steepest descent and 30.000 cycles of conjugate gradients and no restraints were used. The heating and equilibration stage was divided into 14 steps. The temperature was gradually increased until it reached 300 K. Langevin dynamics (thermostat) was employed with a collision frequency of 3.0 ps^−1^. A harmonic potential of 25 kcal mol^−1^ × Å^−2^ was employed in the initial steps and was turned off during step 14. The heating procedure lasted 650 ps until step 14 and was performed using an NVT ensemble. After that, a 2‐ns equilibration phase was employed in an NPT ensemble. The SHAKE algorithm was applied to restrict the vibration of the ligations of all hydrogen atoms. The particle mesh Ewald method was used for calculating electrostatic interactions using a cutoff value of 10.0 Å.

### 2.3 Statistical Analysis

Averages and standard deviation values were calculated for all parameters (RMSD, RMSF, SASA, and *R*_g_) across the triplicate simulations for each system. To evaluate the relative variability among replicates, the coefficient of variation (CV; which is defined as the standard deviation divided by the mean expressed as a percentage) was analyzed for each parameter. A one‐way ANOVA was performed on RMSD values calculated from the last 50 ns of the trajectories (frames 75.000–100.000) to assess the effect of metal type on structural deviation. When significant differences were detected, post hoc pairwise comparisons were conducted using Tukey's test to identify statistically distinct groups.

## 3 Results and Discussion

### 3.1 Moderate‐Quality Metallothionein Model From Foldseek‐Retrieved AlphaFold, Offering Interesting Insights Through Its Flexibility

**FIGURE 1:**
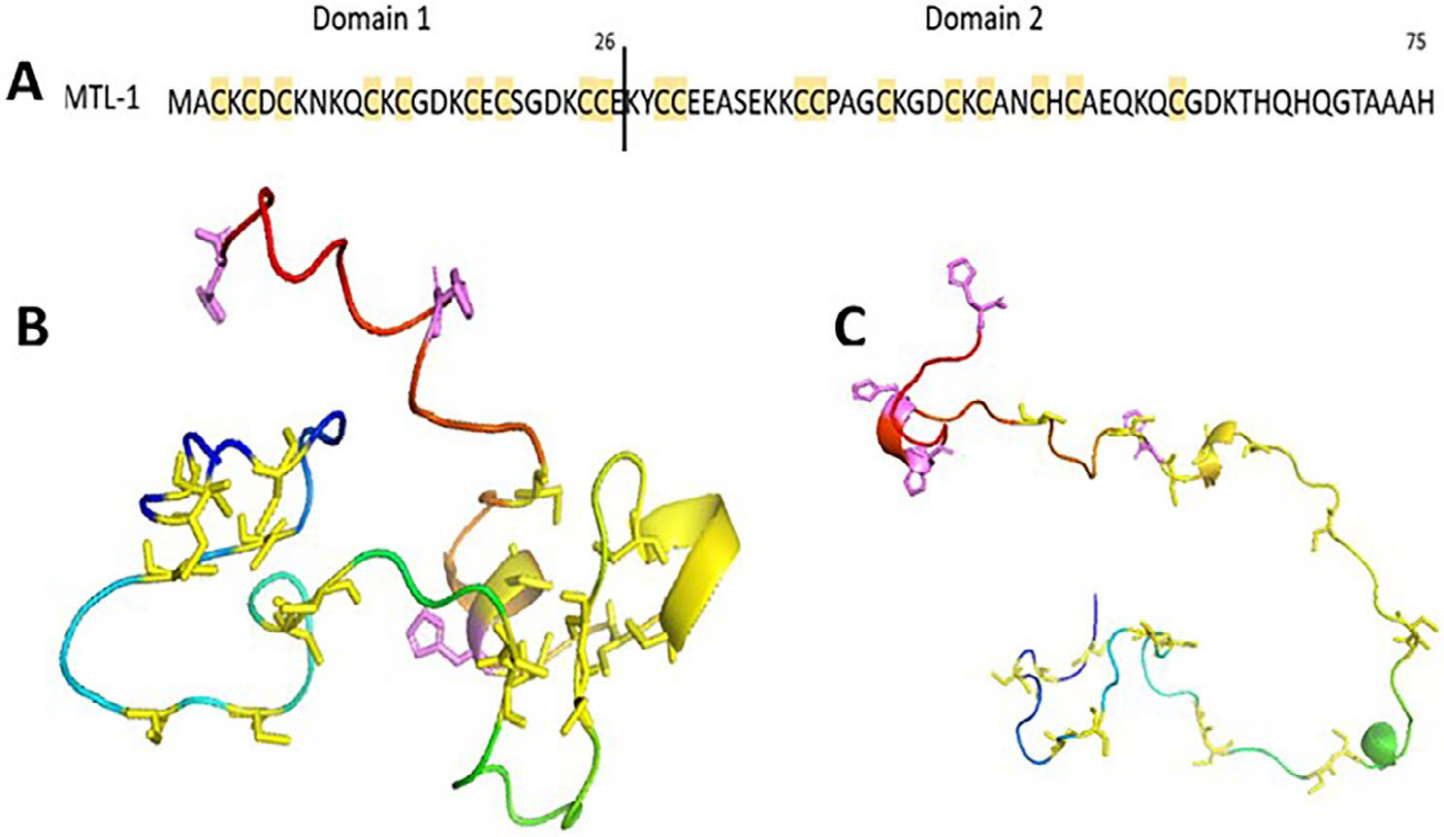
Sequence and 3D structural models of metallothionein I isoform (MTL‐1) of *C. elegans* . (A) Amino acid sequence, with cysteine residues highlighted in yellow. (B) 3D model of MTL‐1 generated by AlphaFold Protein Structure Database (AFDB) using the Foldseek platform, showing deprotonated Cys and His residues as yellow and violet sticks, respectively. (C) Metallothionein without metal (MTL‐1 Apo) after 200 ns of MD simulation, in center specified coordinates onto reference structure, with Cys and His residues highlighted in yellow and violet, respectively.

Modeling small and flexible proteins remains a challenge due to the limited structural information and high conformational plasticity [50, 51]. In this context, recent advances in deep learning‐based approaches such as AlphaFold have offered valuable insights [31, 52]. The three‐dimensional model of MTL‐1 from *C. elegans* was retrieved directly from the AlphaFold Protein Structure Database (AFDB) using the Foldseek platform (P17511) (Figure 1B), which identifies high‐confidence structures based on fold‐level similarity rather than solely sequence homology. This is particularly advantageous for metallothionein, which is a small, cysteine‐rich, and intrinsically disordered protein with few homologs of known structure for this animal group. While AlphaFold2 typically generates multiple models ranked internally by pLDDT scores, the Foldseek method enables the selection of pre‐ranked structures, optimized based not only on local confidence metrics but also on global structural similarity to the entire AlphaFold database [53].

The selected model spans the entire protein sequence (residues 1–75), complementing the limited experimental structural data available for MTL‐1 of *C.elegans*, which includes only a partial NMR structure (PDB:8AQ9). The parameter evaluating the geometry indicated moderate quality, with MolProbity score of 2.35 (MolProbity scores < 2.0 are typical of high‐resolution crystal structures, while values < 3.0 are considered reasonable for predicted models). The GMQE score was 0.60, which also reflects a moderate expected accuracy. GMQE values range from 0 to 1, which higher values indication higher model reliability. Scores around 0.5–0.7 are typical for models based on proteins lacking close structural homologs—conditions applicable to MTL‐1, which has no close experimental template structure available.

The Ramachandran analysis indicated about 98.5% of residues are located in allowed regions, with just 1.5% residues in disallowed regions (Table 1), a result that is generally interpreted as indicative of good stereochemical quality (typically, > 90% in allowed regions is considered acceptable for homology models). Validation with ERRAT resulted in a score of 91%, a value considered high and indicative of a low number of errors in non‐bonded atom‐atom interactions. For context, an ERRAT score above 80% is often considered indicative of reliable structural regions. ProSa analysis yielded a *Z*‐score of 7.23, consistent with proteins of similar size [54]. The QMEAN score of −3.43 suggested structural pattern deviations, particularly in regions predicted with lower confidence. Importantly, the model also presented an average pLDDT score of approximately 60, which corresponds to moderate confidence according to AlphaFold standards. Although this does not indicate high atomic precision, it is expected for proteins like metallothionein, which are intrinsically disordered and rich in flexible, metal‐binding loops. AlphaFold systematically assigns lower pLDDT values to such regions.

**TABLE 1:** Validation of the *C. elegans* metallothionein I model structure using AlphaFold.

Ramachandran analysis	%	pDLLT	Molprobity	GMQE	QMEAN
Residues in most favored	65.2	59.8	2.35	0.60	−3.43
Residues in additional allowed regions	30.3				
Residues in generously allowed regions	3.0				
Residues in disallowed regions	1.5				

The high negative value of QMEAN (−3.43) and moderate value of pLDDT of the MTL‐1 model indicated structural patterns deviation, pointing to low‐confidence regions identified by AlphaFold. Despite that, it has been recognized that proteins with segments of low confidence may contain important information about conditional order, that is, the final protein conformation is not extremely stable but rather is on the edge of stability [55]. This means that the folded state can be easily altered by small variations in environmental conditions (e.g., pH [56] or modifications to the amino acid sequence). However, such changes may not be captured by AlphaFold predictions due to its limitations, such as those arising from partially documented structures or methodological constraints [57]. This behavior is consistent with the properties of intrinsically disordered proteins (IDPs) [55, 58], which is quite common; for example, over 30% of regions within the humane proteome cataloged in AlphaFold are disordered [52]. Therefore, in this context, AlphaFold predictions, which include structural annotations for disordered proteins, can be a valuable resource when experimental protein resolutions (e.g., x‐ray crystallography and nuclear magnetic resonance [NMR]) are absent or difficult to obtain, such as metallothionein.

### 3.2 Metal Coordination Enhances Conformational Stability in Flexible MTL‐1

**FIGURE 2:**
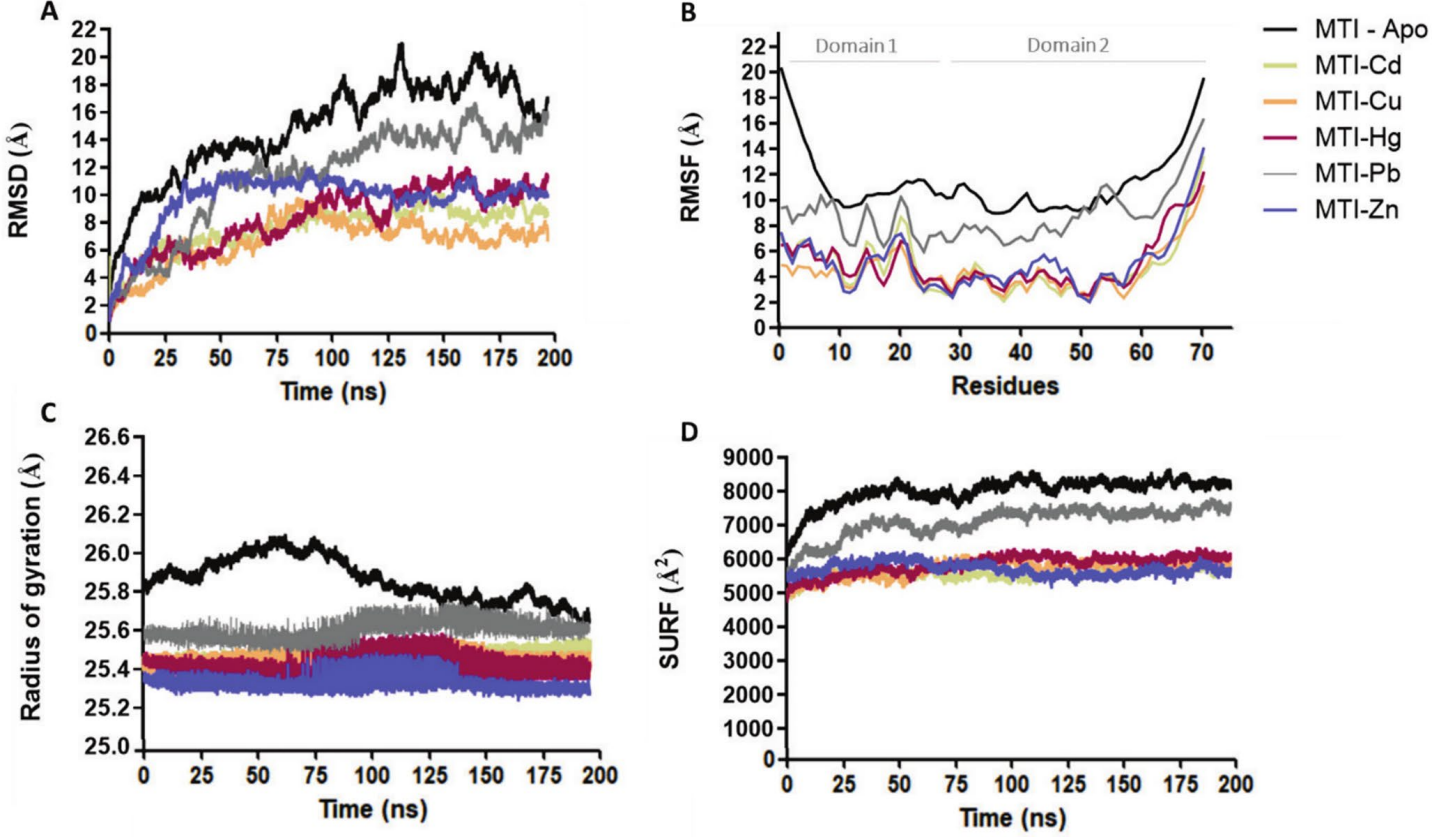
All‐atom molecular dynamics simulations of *C. elegans* metallothionein I isoform in the absence (MTL‐1‐Apo) and coordination of different divalent metal ions. Except for MTL‐1‐Apo, each system is formed by MTI protein and 7 homometallic ions. (A) root mean square deviation (RMSD), (B) root mean square fluctuation (RMSF), (C) radius of gyrations (*R*_g_), and (D) solvent accessible surface area (SASA). Each parameter represents the average of the triplicate simulations.

In this study, the apo form of MTL‐1 was used as a reference structure. Although simulated without metal ions, this apo state was prepared in a coordination‐competent conformation, with deprotonated Cys residues (thiolates, S^−^) and neutral His residues, to reflect a functionally pre‐organized state for potential metal interaction. This protonation scheme ensures reproducibility across apo and holo systems and maintains a conformation suitable for potential metal coordination. While the net negative charge of the apo system results in a more expanded structure, as reflected in the radius of gyration (Figure 2C), the relative spatial arrangement of key coordinating residues is still preserved (Figure 1C). This preparation strategy is supported by previous studies showing that cysteine typically adopts the thiolate form for metal coordination, whereas histidine imidazole, though less frequently observed, has been reported in other metal‐binding proteins and coordination chemistry studies [7, 8, 20, 59, 60, 61, 62, 63]. Modeling these residues as Cys^−^ and neutral His likely promotes a favorable environment for interaction with different divalent metal ions. Although protonation states can vary depending on the local environment, this setup offers a chemically reasonable approximation of potential coordination states. It is consistent with approaches frequently adopted in exploratory studies to examine metal‐binding dynamics under near‐functional conditions [64]. The coefficient of variation (CV) for the simulation triplicates indicated a high degree of reliability and reproducibility. Both SASA and *R*_g_ showed low variability (below 10%), while the RMSD and RMSF displayed moderate variability, but acceptable, supporting the consistency of the simulation results (see Figures S1–S4).

As expected, the MTL systems with and without metal exhibited relatively high flexibility (high values of RMSD, Table 2; Figure 2A) whereas the apo form exhibited the highest RMSD (17.77 ± 1.08 Å), reflecting an extended and flexible protein, if compared to globular structure (commonly 1.0–2.5 Å), consistent with the known structural disorder of metallothioneins [65]. MTL‐1‐Apo exhibited an extended structure (Figure 1C), consisting of approximately 86% turn, bend, and coil conformations (Table S1), which predictably resulted in the highest of molecular dynamic parameters results (Figure 2A–D).

**TABLE 2:** ANOVA and pairwise post hoc analysis for RMSD values comparison across MTL‐1 systems simulations without metals and with metals, in triplicate, over the final 50 ns.

	df	MS	*F*	*p*
MTL‐1 systems	5	1 974 689	686 602	**< 0.001**
Residues	150 000	86 281		

Pos hoc analysis	
Group	*p*
MTL‐1 Cd^2+^ − MTL‐1 Apo	**< 0.001**
MTL‐1 Cu^2+^ − MTL‐1 Apo	**< 0.001**
MTL‐1 Hg^2+^ − MTL‐1 Apo	**< 0.001**
MTL‐1 Pb^2+^ − MTL‐1 Apo	**< 0.001**
MTL‐1 Zn^2+^ − MTL‐1 Apo	**< 0.001**
MTL‐1 Cu^2+^ − MTL‐1 Cd^2+^	1.000
MTL‐1 Hg^2+^ − MTL‐1 Cd^2+^	**< 0.001**
MTL‐1 Pb^2+^ − MTL‐1 Cd^2+^	**< 0.001**
MTL‐1 Zn^2+^ − MTL‐1 Cd^2+^	**< 0.001**
MTL‐1 Hg^2+^ − MTL‐1 Cu^2+^	**< 0.001**
MTL‐1 Pb^2+^ − MTL‐1 Cu^2+^	**< 0.001**
MTL‐1 Zn^2+^ − MTL‐1 Cu^2+^	**< 0.001**
MTL‐1 Pb^2+^ − MTL‐1 Hg^2+^	**< 0.001**
MTL‐1 Zn^2+^ − MTL‐1 Hg^2+^	**< 0.001**
MTL‐1 Zn^2+^ − MTL‐1 Pb^2+^	**< 0.001**

*Note*: Values in bold represent *p* < 0.05.

This outcome corroborates experimental studies by circular dichroism spectroscopy [66, 67, 68] and MD studies [69] that indicate that metallothionein is relatively unstructured in the absence of metal ions, content minimal traditional secondary structural (see Table S1) elements such as α‐helices or β‐pleated sheets prevailing random coil structure [68]. This structural condition of MT‐Apo, observed in different species, appears necessary to precede the cooperative unfolding transition, characterized by a coordinated all‐or‐nothing change in the protein's structure, indicating that this native conformation is crucial for the unfolding process [70, 71]. This observation has broader implications since some studies suggest that MTL‐1 Apo is not simply an unstructured protein. Instead, it is proposed that in physiological conditions, the native structure may adopt folded conformation, contrary to the extended structure [72, 73]. Others suggest that MT‐Apo (2A human MT) samples several conformational states ranging from globular‐like compact to extended conformations [74]. Although these observations provide valuable insights, ongoing studies are needed to fully understand these structural dynamics. Indeed, the biological function of the “denatured‐like structure” of MTL Apo is particularly remarkable. Beyond its crucial role in metal sequestration, it significantly contributes to the protein's interaction with cellular oxidants, underscoring its importance in cellular redox regulation and stress response [75].

Conversely, analysis of the metal‐coordinated systems revealed that Cd^2+^, Zn^2+^, Cu^2+^, and Hg^2+^ coordination stabilized MTI‐1, as indicated by lower RMSD values compared to MTL‐1 Apo (Table 2; Figure 2A). It is important to clarify that “stability” here refers to structural compactions and reduced flexibility observed in the simulations. Our models do not assess thermodynamic stability or quantum effects; thus, reported stability reflects relative conformational instead of energetic stability. More rigorous analysis of MTL‐1 metal‐binding energetics would require advanced methods like quantum calculations, which are beyond this study's scope.

RMSD values over the final 50 ns revealed a highly significant effect of metal type on structural deviation (*F* = 686 602, *p* < 0.001). Post hoc Tukey tests indicated that Cu^2+^ and Cd^2+^ did not differ significantly (*p* = 1.0), indicating similar structural compaction induced by these metals. Both Cu^2+^ and Cd^2+^ differed significantly from Zn^2+^ and Hg^2+^ (*p* < 0.001) which also differed among themselves. Pb^2+^ exhibited the highest RMSD (11.04 ± 0.60 Å) and differed significantly from all other metals (*p* < 0.001). Average RMSD values were: 6.73 ± 0.60 Å for Hg^2+^; 6.99 ± 0.35 Å for Cu^2+^; 8.76 ± 0.37 Å for Cd^2+^; and 10.11 ± 0.39 Å for Zn^2+^ (Figure 2A). These results indicate that metal coordination stabilizes MTL‐1 in a metal‐dependent manner, with Zn^2+^, Cd^2+^, Cu^2+^, and Hg^2+^ favoring compact, stable structures, whereas Pb^2+^ leads to increased flexibility and reduced structural order. This same pattern was visually observed for the other parameters analyzed (see Figure 2B–D). Experimental data have revealed that the presence of metals induces the formation of a more ordered and stable structure and stabilizes MTL protein structure through coordination interaction. This stabilization has been observed in various organisms, including mammals [76], plants [28, 77], earthworms [15], fungi [78], and bacteria [79]. Although different isoforms exhibit varying affinities, laboratory data for *C. elegans* MTL‐1 specifically demonstrated that this isoform folds more effectively in the presence of zinc than cadmium [9]. Our simulation data align with this observation, as the Zn^2+^ system showed slightly lower values for the radius of gyration (Figure 2C). This parameter represents changes in protein compaction reflecting the distribution of mass around the center of mass. Despite the small variation, this may indicate a tendency toward compaction induced by Zn^2+^ metal, suggesting a more compact and stable structure, even if not causing a drastic structural change. On the other hand, Pb^2+^, in particular, did not confer the same structural stability as the other metals tested, as evidenced by higher RMSD (Table 2). The SASA and radius of gyration value profiles for the Pb^2+^ system also displayed relatively higher values (Figure 2) indicating a more flexible and dynamic conformation compared to other metal‐coordination systems (Figure 2). This suggests that Pb^2+^ leads to a less compact structure, jeopardizing the overall stability of the protein. Although there is experimental evidence that Cys residues from MTL can bind to Pb^2+^ [80], this process is quite sensitive to other factors, which will be further discussed in the next section. Experimental studies exploring the interaction of MTL‐1 with Cu^2+^ and Hg^2+^ are limited. Therefore, our simulation study sought to address this gap by investigating the coordination and structural stability of MTL‐1 in the presence of these metals.

The RMSF data revealed that metal coordination of Cd^2+^, Zn^2+^, Cu^2+^, and Hg^2+^ induced similar patterns in the variation of residue flexibility, with only a slight distinction in fluctuation intensity, except for Pb^2+^ (Figure 2B). This means that the most flexible and most rigid regions of the protein remain consistent, regardless of the metal coordination, which may indicate a structured global function of this protein when exposed to metal. In this context, the selectivity of MTL for a specific metal may not strongly depend on the conformation of the protein but on other local factors, such as the metal affinity and the chemistry of the metal involved. The most significant changes were observed in the region near the N‐terminal, particularly at residues Asp16, Lys17, Gly21, and Asp22 (Figure 2B). Regions closest to the C‐terminus of a protein can also exhibit higher RMSF values, especially if the C‐terminus is not involved in stable secondary structures or without interactions. The residues that are more flexible are often located in the loop regions of proteins [81]. In addition, glycine, due to its small size and flexibility, promotes mobility. Meanwhile, lysine, with its long positively charged side chain, and aspartic acid, with its negatively charged carboxyl group, further enhance dynamics and flexibility by interacting with the solvent.

Analysis of the apo form (Figure 2B) revealed even higher fluctuations, particularly in the metal‐coordination regions. While overall more dynamic, these fluctuations follow non‐random, localized patterns, suggesting that structural elements involved in coordinating are partially pre‐organized [14, 82]. This likely facilitates the rapid stabilization observed upon coordination of Cd^2+^, Cu^2+^, Zn^2+^, and Hg^2+^. The limited reduction in flexibility seen with Pb^2+^ further emphasizes the importance of metal‐specific compatibility.

Furthermore, considering the MTL‐1 fold in *C. elegans* , biophysical studies have proposed a two‐domain model for MTL‐1 [41], with domain 1 comprising residues 1–26 and domain 2 comprising 27–75. Similarly, mammalian MTL‐3 has a two‐domain structure, and experimental studies indicated that the β‐domain (residues 1–31) is structurally more flexible, while the α‐domain (residues 32–68) is more stable [83]. Although the primary protein sequences of mammalian MTL‐3 and *C. elegans* MTL‐1 are dissimilar, MTL‐1 showed similar behavior, exhibiting higher flexibility residues in domain I, with peaks and higher absolute value of RMSF when compared to domain 2 (Figure 2B). Therefore, our results align with these findings, indicating that the flexibility patterns of MT in *C. elegans* mirror the established structural characteristics of mammalian metallothionein isoform. This similarity may suggest that the three‐dimensional behavior of the protein structure may be conserved across species.

The consistence interdependence among these metrics—RMSD, RMSF, radius of gyration, and SASA—demonstrates the overall stability effects that the metal coordination causes to structure MTL‐1. The SASA variation, ranging from 5000 to 6000 Å^2^ in the Cd^2+^, Zn^2+^, Cu^2+^, and Hg^2+^ systems, does not indicate big changes in the exposure of the protein's surface to the water solvent. Similarly, the minimal variation of the radius of gyration (approximately 0.3 Å) did not lead to drastic shifts in the compaction or extension of MTL‐1. The results for both parameters suggest that the overall conformation of metallothionein is maintained similarly among the different metals, and small differences are likely related to specific interactions with the coordinating residues, or the specific characteristics of each metal.

### 3.3 Variability and Influence of Distinct Metal Ions in MTL‐1 Coordination

**FIGURE 3:**
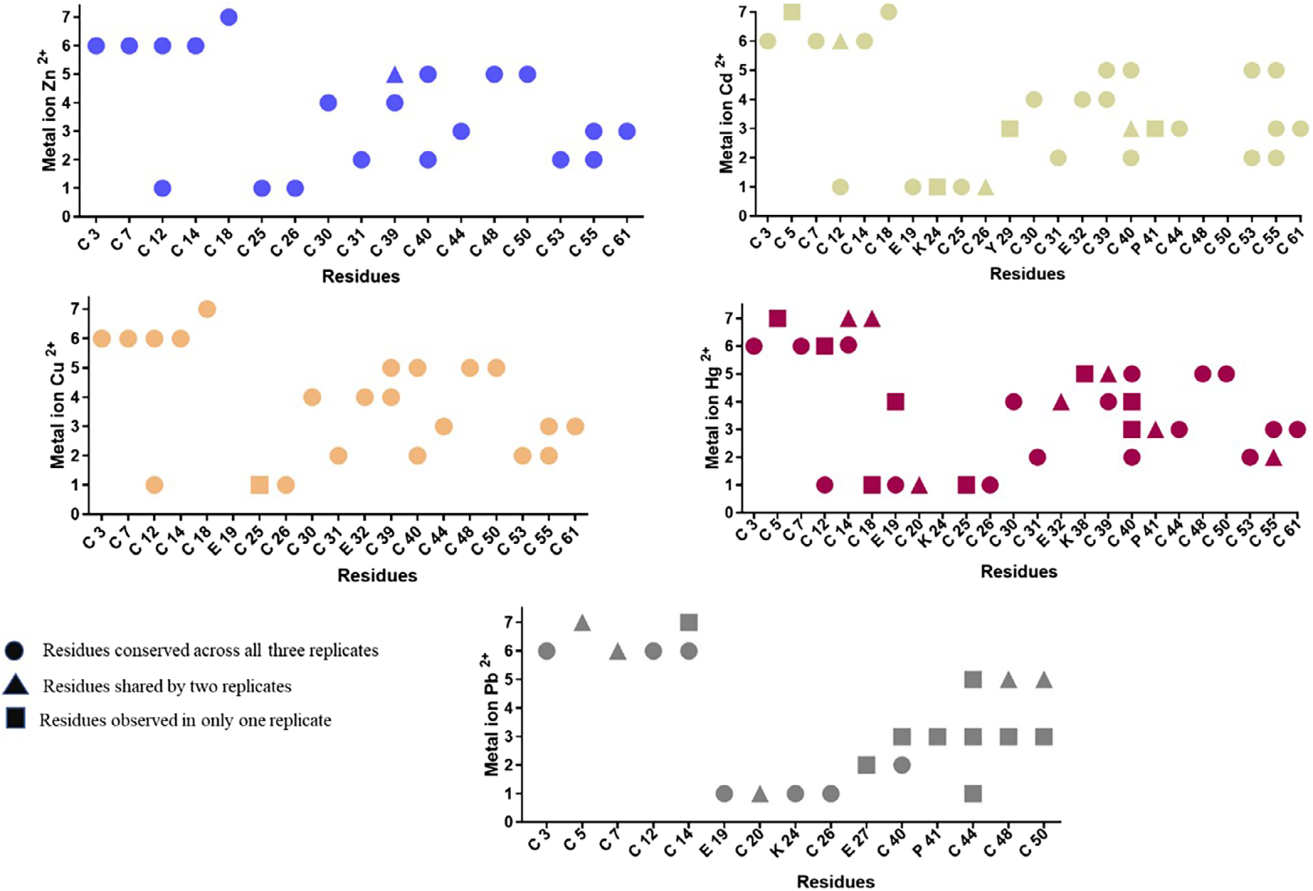
Residues involved in the coordination site of each MTL‐1 system with seven ions from the divalent metals three simulation replicates (A) Zn, (B) Cd, (C) Cu, (D) Hg, and (E) Pb. Residues on the x‐axis are represented by single‐letter amino acid codes of MTL‐1 sequence: C = cysteine, D = aspartic acid, E = glutamic acid, K = lysine, P = proline, and Y = tyrosine.

The putative coordination sites were inferred based on stable proximity between the metal ions and amino acids. The distances among them were < 2.0 Å for Cu^2+^, < 2.5 Å for Zn^2+^, Cd^2+^, and Hg^2+^, and < 3.0 Å for Pb^2+^ (see Figures S5–S9). Distances below 3.0 Å indicate strong coordination bonds. As predicted, cysteine was the most frequently involved amino acid in these interactions, forming stable complexes with the metal ions. The coordination site structure remained largely unchanged when substituting Zn^2+^, Cd^2+^, Co^2+^, or Hg^2+^, except for Pb^2+^ (Figure 3). Cysteine is essential for the function of metallothionein as a metal sequester with certain cysteine positions playing a more important role in the metal interaction and structuration of MTL [69]. Here, residues such as Cys12, Cys26, Cys31, Cys40, and Cys55 were often involved in coordination with tested metals, which suggests that they are fundamental to the structure of MTL‐1, being essential for effective metal sequestration (Figure 3).

**FIGURE 4:**
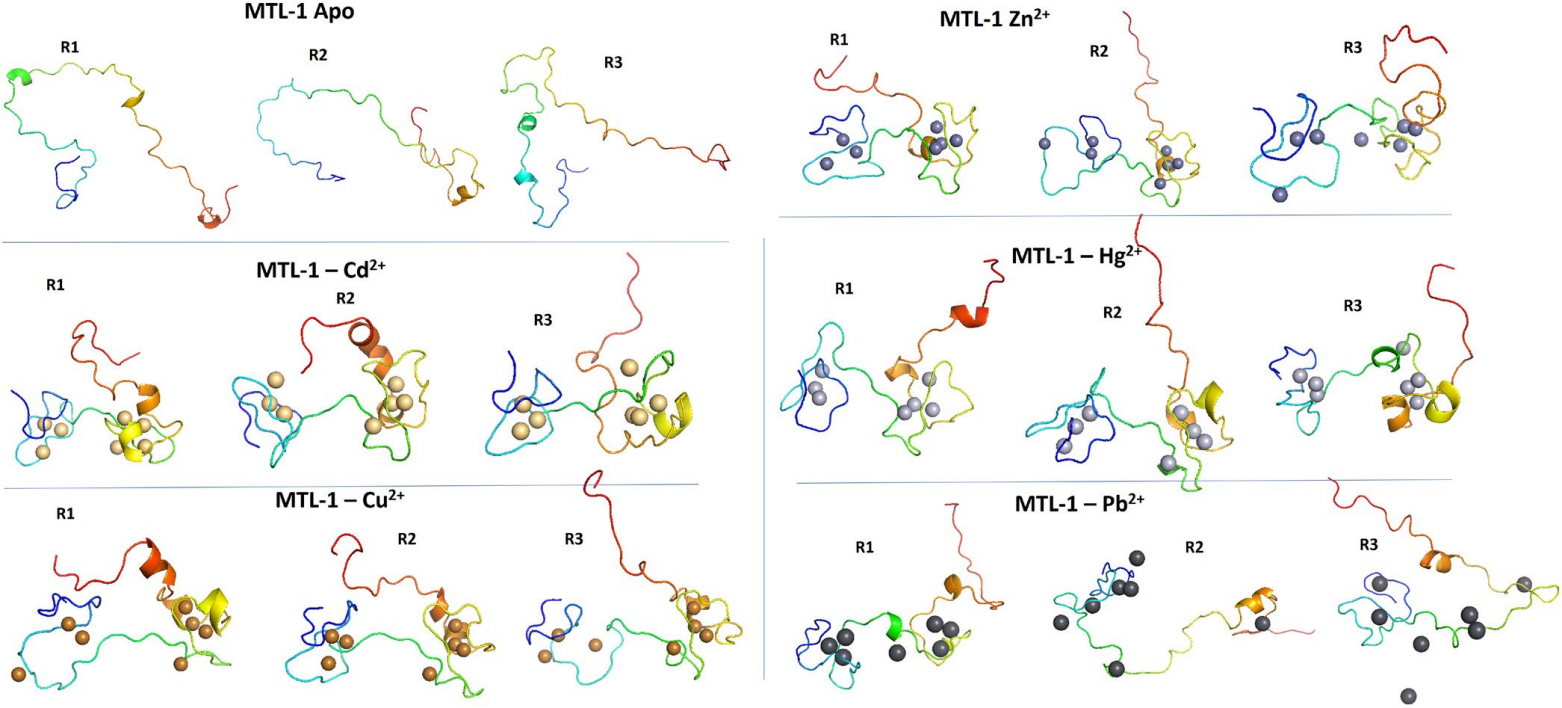
Three‐dimensional structure of MTL‐1 triplicates in the Apo form (without metal) and complexes with five different divalent metals (Zn^2+^, Cd^2+^, Hg^2+^, Cu^2+^, and Pb^2+^) obtained from molecular dynamics simulation (200 ns) using the AMBER software. The structures were visualized using PyMOL. Simulations were performed in three independent replicas (R1, R2, and R3).

In our study, all metal complexes showed some level of clustering or compaction throughout the simulation that is characteristic of cluster structures. At the same time, Pb^2+^ presented a more intermediate distribution, aligning more with the beaded‐like structure (Figure 4). In experimental condition, the compact arrangement involves bridging thiolates, that is, the sulfur in cysteine forms a “bridge” between two metals ions, coordinating with both and thereby linking them together, which herein engaged Cys12, Cys39, Cys40, and Cys55 residues participation especially on the coordination Zn^2+^, Cd^2+^, Cu^2+^, and Hg^2+^ metal. The clustering organization is a key feature of the cooperative metalation pathway proposed for human MTLs [66, 84], in which metalation is facilitated to promote protein structuring. On the other hand, non‐cooperativity occurs when one metalation event does not facilitate the subsequent metalation events. Although we just tested the full metalation, without evaluate the effects of ion addition, we observed that Pb^2+^ metal set did not fit well into the coordination environment of metallothionein and did not follow the typical co‐operative pattern of other metals used. The non‐cooperative behavior of Pb^2+^ may have led to a more disordered or flexible MTL structure, with significant variations in ion positions and protein conformation, which can be observed in the simulation as a greater variation in RMSD and radius of gyration, indicating a less stable structure (Figure 2).

Global stability in metallothionein proteins is influenced by various factors, including physicochemical conditions [85], but metal characteristics are particularly important. Generally, the affinity of a divalent metal ion for a specific ligand (or set of ligands) is correlated with the metal's second ionization enthalpy [86]. This correlation follows the Irving–Williams series [87], which ranks the relative stability of complexes formed by divalent metal ions in the following order: Mg^2+^ < Mn^2+^ < Fe^2+^ < Co^2+^ < Ni^2+^ < Cu^2+^ > Zn^2+^. In our study, after the general convergence of systems (Figure 2), Cu^2+^ had the highest MTL‐1 stability considering the RMSD value parameter, followed by Zn^2+^, Hg^2+^, and Cd^2+^ with slight differences. The same scenario was observed in another study which proposed the order of metal ion affinity to Cys residue, Cu > Zn > Hg > Cd, using density functional theory calculations [88]. On the other hand, Pb^2+^ ions caused the lowest MTL stability, leading to higher RMSD values (Figure 2A).

In experimental conditions, Pb^2+^ coordination to MTL is characterized by a high degree of variability and complexity, with some complexes becoming more stable while others may dissociate and become highly sensitive to pH changes when compared to other metals [89, 90]. This could partially explain the diversity of conformer variations in the simulations for this metal. Deviations were observed in the coordination sites for some Pb^2+^ metal across simulation runs. Specifically, ions 4 and 7 consistently lacked coordination with the protein—ion 4 in all replicates, and ion 7 in replicate 3. Additionally, ions 3 and 5 exhibited relatively weak and inconsistent coordination with residues in replicates 2 and 3 (Figure S9). This lack of or weak coordination was not observed with any other metal tested. The affinity of Pb^2+^ for MTL can be relatively weak compared to other metals like zinc or cadmium. This is primarily due to the nature of the interactions involved. Studies have shown that Pb^2+^ binding to MTL is less stable, leading to a higher likelihood of dissociation under physiological conditions [60]. In addition, Cys residues remained the primary coordination sites for Pb with MTL‐1, where an absence of bridging cysteine sulfur was observed, a feature that has previously been associated with increased flexibility in Pb_7_‐MT2(II) from rabbit liver [91].

Furthermore, while in our simulation, the deprotonation of all cysteines in the MTL system with Pb^2+^ may have favored the affinity of Pb^2+^ ions for the deprotonated thiolate ligands, potentially increasing the stability of the complex, it could also promote changes in electrostatic interactions and coordination geometry of the protein. Finally, the coordination of Pb^2+^ with MT can have a time‐dependent behavior, which may vary over time due to the unique coordination properties of metal [92]. This could influence the stability and conformation of the metallothionein complex, leading to potential variations in coordination site occupancy and overall protein structure during molecular dynamics simulations. In this context, further deep studies are required to understand the exact mechanisms governing the unique behavior of Pb^2+^ in metallothionein complexes.

### 3.4 Metal Interactions in MTL‐1: Cysteines and Beyond

Cys residues allow the formation of high‐affinity complexes with metals, forming “anchor points,” which can be crucial for protein function, at first. However, versatility in coordination with metals may be a crucial aspect of the function of IDPs. The coordination sites on metallothionein can be highly dynamic and capable of adapting rapidly to different metals, which can be essential for the regulation and function of this protein in response to variable conditions. The increased presence of cysteines in MTL sequences also correlates with increased disorder propensity within the sequences that flank the cysteines, which potentially increases conformational plasticity under reducing conditions, such as our simulation condition [93]. In this context, besides cysteine, other residues may play a crucial role in metal coordination. In our study, Glu residues were particularly important for coordinating Cd and Hg ions along with Cys (see Figures S6 and S8). Additionally, Glu was involved in coordinating one of the Cu ions. Lys, Tyr, and Pro also emerged as bond‐stabilizing residues, with Lys participating in the coordination of Pb. These non‐Cys residues maintained a distance of less than 2.5 Å throughout the entire simulation, indicating strong coordination interaction between metals and amino acid residues in MTL‐1.

In relation to the diverse coordination roles observed for non‐Cys, it is observed that histidine residues, despite being modeled in their neutral form (HIE), did not participate in stable metal coordination in any of the simulated systems. This suggests that for MTL‐1 and the metals studied, the spatial arrangement of the histidine side chains [94] and the metal's stronger preference for cysteine thiolates outweigh their potential coordinative capability under these conditions [95].

Experimental and modeling studies have been providing evidence that other residues such as Glu, Lys, Ser, and Asp can participate in metal coordination together with cysteines [69, 96, 97] affecting the modulation of the complex's structure and stability. The specific coordination of non‐Cys residues with metals can be attributed to a combination of factors, such as specific electrostatic interactions or the local conformation. The Glu residue, for example, has a carboxylate group (—COO^−^), which can interact with metals to make the bond more stable, even if it is not the first choice in terms of chemical affinity. This residue at positions 19 and 32, flanked by two nearby Cys residues each, may have altered the affinity of the metal ions [96], especially for Hg^2+^ which has a high polarizability; that means its electron cloud can be easily distorted by an external electric field. The presence of non‐Cys residues in MTL‐1 is as crucial as the presence of Cys, as these non‐Cys residues can influence which metals are preferentially coordinated and how effectively they are chelated [98]. Furthermore, other explanatory factors, such as the size and geometry of the interaction site cavity, are also important aspects that can help in understanding the selectivity of the metal ion [46].

As mentioned, Pb^2+^ was observed to induce the least stability among the metals tested, showing varied interaction patterns across different residues. Experimental studies appoint indirect evidence that the coordination of Pb^2+^ in thiol‐rich environments, such as glutathione and zinc fingers, is consistent with the preference of Pb^2+^ for strong coordination with sulfur atoms, which are functional analogues to the interactions observed in your simulated metallothionein [99]. Confirming, Pb^2+^ coordinates strongly with some cysteine residues (Figure S9). On the other hand, the coordination chemistry of Pb^2+^, together with the expanded conformation and dynamics of the protein, seems to favor a condition for a transient interaction with non‐Cys residues. Indeed, studies on lead coordination to metallothionein indicate that the metalation process varies with different experimental conditions [90]. Furthermore, although MTL plays a role in the coordination and detoxification of Pb^2+^, its capacity and efficiency in this function are less responsive to lead than to other metals such as Zn and Cd [100]. These characteristics, such as a high rate of associated changes or moderate affinities at specific coordination sites, are common in IDP interactions. These interactions are likely a combination of conformational selection and induced fit mechanisms, relying on existing structural elements and structural changes after binding [101].

## 4 Conclusions

This study provides valuable insights into the structural flexibility and metal‐coordination properties of *C. elegans* metallothionein I (MTL‐1) using molecular dynamics simulations and AlphaFold‐based modeling. Despite moderate confidence in some structural predictions due to the intrinsically disordered nature of MTL‐1, the simulations reveal that metal coordination significantly stabilizes the protein structure, with Zn^2+^ (*R*_g_) and Cu^2+^ (RMSD) inducing the highest levels of compaction and stability. The exception was Pb^2+^, which showed weaker stabilization, suggesting non‐cooperative coordination and greater flexibility in the protein structure. Although our approach provides useful structural insights, it does not fully capture thermodynamic or quantum mechanical aspects of metal binding, which could be addressed in future studies. The role of non‐cysteine residues in metal coordination observed here underscores the complex modulation of metallothionein function that extends beyond classical cysteine–metal interactions. These findings not only confirm previous experimental observations about MTL's dependence on metal coordination but also provide new insights into the structural dynamics of metallothionein in response to different metals.

## Author Contributions

**Nilvea Ramalho de Oliveira:** conceptualization, methodology, investigation, formal analysis, visualization, writing – original draft. **Andrei Santos Siqueira:** methodology, writing – review and editing, investigation, formal analysis, visualization. **Paulo Sérgio Alves Bueno:** writing – review and editing, formal analysis, visualization, methodology. **Evonnildo Costa Gonçalves:** writing – review and editing, resources. **Juliano Zanette:** supervision, writing – review and editing, conceptualization, funding acquisition, investigation.

## Supporting information


**Data S1:** Supporting Information.

## Data Availability

The data that support the findings of this study are available in the Supporting Information of this article.

## References

[1] B. Berthet , C. Mouneyrac , T. Pérez , and C. Amiard‐Triquet , “Metallothionein Concentration in Sponges ( *Spongia officinalis* ) as a Biomarker of Metal Contamination,” Comparative Biochemistry and Physiology Part C: Toxicology & Pharmacology 141, no. 3 (2005): 306–313, 10.1016/j.cca.2005.07.008. 16098817

[2] M. Capdevila , R. Bofill , Ò. Palacios , and S. Atrian , “State‐of‐the‐Art of Metallothioneins at the Beginning of the 21st Century,” Coordination Chemistry Reviews 256, no. 1–2 (2012): 46–62, 10.1016/j.ccr.2011.07.006.

[3] G. Isani and E. Carpenè , “Metallothioneins, Unconventional Proteins From Unconventional Animals: A Long Journey From Nematodes to Mammals,” Biomolecules 4, no. 2 (2014): 435–457, 10.3390/biom4020435. 24970224 PMC4101491

[4] C. A. Blindauer and O. I. Leszczyszyn , “Metallothioneins: Unparalleled Diversity in Structures and Functions for Metal Ion Homeostasis and More,” Natural Product Reports 27, no. 5 (2010): 720–741, 10.1039/b906685n. 20442962

[5] N. Thirumoorthy , K. T. M. Kumar , A. S. Sundar , L. Panayappan , and M. Chatterjee , “Metallothionein: An Overview,” World Journal of Gastroenterology 13, no. 7 (2007): 993–996. 17373731 10.3748/wjg.v13.i7.993 PMC4146885

[6] M. Imagawa , T. Onozawa , K. Okumura , S. Osada , T. Nishihara , and M. Kondo , “Characterization of Metallothionein cDNAs Induced by Cadmium in the Nematode *Caenorhabditis elegans* ,” Biochemical Journal 268, no. 1 (1990): 237–240, 10.1042/bj2680237. 2344361 PMC1131418

[7] C. A. Blindauer , “Metallothioneins With Unusual Residues: Histidines as Modulators of Zinc Affinity and Reactivity,” Journal of Inorganic Biochemistry 102, no. 3 (2008): 507–521, 10.1016/j.jinorgbio.2007.10.032. 18171588

[8] R. Bofill , R. Orihuela , M. Romagosa , J. Domènech , S. Atrian , and M. Capdevila , “ *Caenorhabditis elegans* Metallothionein Isoform Specificity—Metal Binding Abilities and the Role of Histidine in CeMT1 and CeMT2,” FEBS Journal 276, no. 23 (2009): 7040–7056, 10.1111/j.1742-4658.2009.07417.x. 19860833

[9] Y. J. Essig , O. I. Leszczyszyn , N. Almutairi , et al., “Juggling Cadmium Detoxification and Zinc Homeostasis: A Division of Labour Between the Two *C. elegans* Metallothioneins,” Chemosphere 350, no. 2023 (2024): 141021, 10.1016/j.chemosphere.2023.141021. 38151062 PMC11134313

[10] G. C. T. Jiang , S. Hughes , S. R. Stürzenbaum , L. Evje , T. Syversen , and M. Aschner , “ *Caenorhabditis elegans* Metallothioneins Protect Against Toxicity Induced by Depleted Uranium,” Toxicological Sciences 111, no. 2 (2009): 345–354, 10.1093/toxsci/kfp161. 19617453

[11] P. Aggett , G. F. Nordberg , and M. Nordberg , Essential Metals: Assessing Risks From Deficiency and Toxicity, 4th ed. (Elsevier, 2014), 10.1016/B978-0-444-59453-2.00014-7.

[12] A. Beil , S. Jurt , R. Walser , et al., “The Solution Structure and Dynamics of cd‐Metallothionein From *Helix pomatia* Reveal Optimization for Binding cd Over Zn,” Biochemistry 58, no. 45 (2019): 4570–4581, 10.1021/acs.biochem.9b00830. 31633358

[13] P. Chaudhuri , H. T. Imam , Y. Essig , et al., “Molecular Genetic and Biochemical Characterization of a Putative Family of Zinc Metalloproteins in: *Caenorhabditis elegans* ,” Metallomics 10, no. 12 (2018): 1814–1823, 10.1039/c8mt00169c. 30444224 PMC6336089

[14] G. W. Irvine , T. B. J. Pinter , and M. J. Stillman , “Defining the Metal Binding Pathways of Human Metallothionein 1a: Balancing Zinc Availability and Cadmium Seclusion,” Metallomics 8, no. 1 (2016): 71–81, 10.1039/C5MT00225G. 26583802

[15] G. R. Kowald , S. R. Sturzenbaum , and C. A. Blindauer , “Earthworm *Lumbricus Rubellus* MT‐2: Metal Binding and Protein Folding of a True Cadmium‐MT,” International Journal of Molecular Sciences 17, no. 1 (2016): 1–16, 10.3390/ijms17010065. PMC4730310 26742040

[16] J. Hall , K. L. Haas , and J. H. Freedman , “Role of MTL‐1, MTL‐2, and CDR‐1 in Mediating Cadmium Sensitivity in *Caenorhabditis elegans* ,” Toxicological Sciences 128, no. 2 (2012): 418–426, 10.1093/toxsci/kfs166. 22552775 PMC3493192

[17] S. Zeitoun‐Ghandour , J. M. Charnock , M. E. Hodson , O. I. Leszczyszyn , C. A. Blindauer , and S. R. Stürzenbaum , “The Two *Caenorhabditis elegans* Metallothioneins (CeMT‐1 and CeMT‐2) Discriminate Between Essential Zinc and Toxic Cadmium,” FEBS Journal 277, no. 11 (2010): 2531–2542, 10.1111/j.1742-4658.2010.07667.x. 20553489

[18] R. Trivedi and H. A. Nagarajaram , “Intrinsically Disordered Proteins: An Overview,” International Journal of Molecular Sciences 23, no. 22 (2022): 1–30, 10.3390/ijms232214050. PMC9693201 36430530

[19] F. Lermyte , “Roles, Characteristics, and Analysis of Intrinsically Disordered Proteins: A Minireview,” Life 10, no. 12 (2020): 320, 10.3390/life10120320. 33266184 PMC7761095

[20] J. Habjanič , O. Zerbe , and E. Freisinger , “A Histidine‐Rich Pseudomonas Metallothionein With a Disordered Tail Displays Higher Binding Capacity for Cadmium Than Zinc,” Metallomics 10, no. 10 (2018): 1415–1429, 10.1039/c8mt00193f. 30191219

[21] P. Faller , C. Hureau , and G. La Penna , “Metal Ions and Intrinsically Disordered Proteins and Peptides: From cu/Zn Amyloid‐β to General Principles,” Accounts of Chemical Research 47, no. 8 (2014): 2252–2259, 10.1021/ar400293h. 24871565

[22] H. Xie , S. Vucetic , L. M. Iakoucheva , et al., “Functional Anthology of Intrinsic Disorder. 3. Ligands, Post‐Translational Modifications, and Diseases Associated With Intrinsically Disordered Proteins,” Journal of Proteome Research 6, no. 5 (2007): 1917–1932, 10.1021/pr060394e. 17391016 PMC2588348

[23] C. A. Scarff , K. Thalassinos , G. R. Hilton , and J. H. Scrivens , “Travelling Wave Ion Mobility Mass Spectrometry Studies of Protein Structure: Biological Significance and Comparison With X‐Ray Crystallography and Nuclear Magnetic Resonance Spectroscopy Measurements,” Rapid Communications in Mass Spectrometry 22, no. 20 (2008): 3297–3304, 10.1002/rcm.3737. 18816489

[24] A. Prestel , K. Bugge , L. Staby , R. Hendus‐Altenburger , and B. B. Kragelund , “Characterization of Dynamic IDP Complexes by NMR Spectroscopy,” in Methods in Enzymology, vol. 611, 1st ed. (Elsevier Inc, 2018), 193–226, 10.1016/bs.mie.2018.08.026. 30471688

[25] M. D. Peris‐Díaz , R. Guran , C. Domene , et al., “An Integrated Mass Spectrometry and Molecular Dynamics Simulations Approach Reveals the Spatial Organization Impact of Metal‐Binding Sites on the Stability of Metal‐Depleted Metallothionein‐2 Species,” Journal of the American Chemical Society 143, no. 40 (2021): 16486–16501, 10.1021/jacs.1c05495. 34477370 PMC8517974

[26] S. P. Walker , V. V. B. Yallapragada , and M. Tangney , “Arming Yourself for the In Silico Protein Design Revolution,” Trends in Biotechnology 39, no. 7 (2021): 1–14, 10.1016/j.tibtech.2020.10.003. 33139074

[27] M. K. Gupta and R. Vadde , “Insights Into the Structure–Function Relationship of Both Wild and Mutant Zinc Transporter ZnT8 in Human: A Computational Structural Biology Approach,” Journal of Biomolecular Structure and Dynamics 38, no. 1 (2020): 137–151, 10.1080/07391102.2019.1567391. 30633652

[28] G. Singh , S. Tripathi , K. Shanker , and A. Sharma , “Cadmium‐Induced Conformational Changes in Type 2 Metallothionein of Medicinal Plant *Coptis japonica*: Insights From Molecular Dynamics Studies of Apo, Partially and Fully Metalated Forms,” Journal of Biomolecular Structure and Dynamics 37, no. 6 (2019): 1520–1533, 10.1080/07391102.2018.1461688. 29624115

[29] L. Zhang , J. Wu , X. Wang , B. Liu , and B. Ma , “Isolation of Metallothionein Genes and In Silico Structural Characterization of Their Proteins Using Molecular Modeling From Yak ( *Bos grunniens* ),” Biochemical Genetics 50, no. 7–8 (2012): 585–599, 10.1007/s10528-012-9503-7. 22399135

[30] A. Schmidt , M. Hagen , E. Schütze , A. Schmidt , and E. Kothe , “In Silico Prediction of Potential Metallothioneins and Metallohistins in Actinobacteria,” Journal of Basic Microbiology 50, no. 6 (2010): 562–569, 10.1002/jobm.201000055. 21077111

[31] M. Varadi , D. Bertoni , P. Magana , et al., “AlphaFold Protein Structure Database in 2024: Providing Structure Coverage for Over 214 Million Protein Sequences,” Nucleic Acids Research 52, no. D1 (2024): D368–D375, 10.1093/nar/gkad1011. 37933859 PMC10767828

[32] J. Jumper , R. Evans , A. Pritzel , et al., “Highly Accurate Protein Structure Prediction With AlphaFold,” Nature 596, no. 7873 (2021): 583–589, 10.1038/s41586-021-03819-2. 34265844 PMC8371605

[33] P. Benkert , M. Biasini , and T. Schwede , “Toward the Estimation of the Absolute Quality of Individual Protein Structure Models,” Bioinformatics 27, no. 3 (2011): 343–350, 10.1093/bioinformatics/btq662. 21134891 PMC3031035

[34] C. J. Williams , J. J. Headd , N. W. Moriarty , et al., “MolProbity: More and Better Reference Data for Improved All‐Atom Structure Validation,” Protein Science 27, no. 1 (2018): 293–315, 10.1002/pro.3330. 29067766 PMC5734394

[35] C. Colovos and T. O. Yeates , “Verification of Protein Structures: Patterns of Nonbonded Atomic Interactions,” Protein Science 2, no. 9 (1993): 1511–1519, 10.1002/pro.5560020916. 8401235 PMC2142462

[36] M. Wiederstein and M. J. Sippl , “ProSA‐Web: Interactive Web Service for the Recognition of Errors in Three‐Dimensional Structures of Proteins,” Nucleic Acids Research 35, no. Web Server (2007): W407–W410, 10.1093/nar/gkm290. 17517781 PMC1933241

[37] W. Kabsch and C. Sander , “Dictionary of Protein Secondary Structure: Pattern Recognition of Hydrogen‐Bonded and Geometrical Features,” Biopolymers 22, no. 12 (1983): 2577–2637, 10.1002/bip.360221211. 6667333

[38] M. Belcastro , T. Marino , N. Russo , and M. Toscano , “The Role of Glutathione in Cadmium Ion Detoxification: Coordination Modes and Binding Properties—A Density Functional Study,” Journal of Inorganic Biochemistry 103, no. 1 (2009): 50–57, 10.1016/j.jinorgbio.2008.09.002. 18951636

[39] M. Enescu , J. P. Renault , S. Pommeret , J. C. Mialocq , and S. Pin , “Ab Initio Study of cd‐Thiol Complexes: Application to the Modelling of the Metallothionein Active Site,” Physical Chemistry Chemical Physics 5, no. 17 (2003): 3762–3767, 10.1039/b306790d.

[40] D. A. Case , H. M. Aktulga , K. Belfon , et al., “AmberTools,” Journal of Chemical Information and Modeling 63, no. 20 (2023): 6183–6191, 10.1021/acs.jcim.3c01153. 37805934 PMC10598796

[41] S. Zeitoun‐Ghandour , O. I. Leszczyszyn , C. A. Blindauer , F. M. Geier , J. G. Bundy , and S. R. Stürzenbaum , “ *C. elegans* Metallothioneins: Response to and Defence Against ROS Toxicity,” Molecular BioSystems 7, no. 8 (2011): 2397–2406, 10.1039/c1mb05114h. 21647514

[42] O. I. Leszczyszyn , S. Zeitoun‐Ghandour , S. R. Stürzenbaum , and C. A. Blindauer , “Tools for Metal Ion Sorting: In Vitro Evidence for Partitioning of Zinc and Cadmium in *C. elegans* Metallothionein Isoforms,” Chemical Communications 47, no. 1 (2011): 448–450, 10.1039/c0cc02188a. 20877848

[43] J. Wang , R. M. Wolf , J. W. Caldwell , P. A. Kollman , and D. A. Case , “Development and Testing of a General Amber Force Field,” Journal of Computational Chemistry 25, no. 9 (2004): 1157–1174, 10.1002/jcc.20035. 15116359

[44] C. Tian , K. Kasavajhala , K. A. A. Belfon , et al., “Ff19SB: Amino‐Acid‐Specific Protein Backbone Parameters Trained Against Quantum Mechanics Energy Surfaces in Solution,” Journal of Chemical Theory and Computation 16, no. 1 (2020): 528–552, 10.1021/acs.jctc.9b00591. 31714766 PMC13071887

[45] S. Izadi , R. Anandakrishnan , and A. V. Onufriev , “Building Water Models: A Different Approach,” Journal of Physical Chemistry Letters 5, no. 21 (2014): 3863–3871, 10.1021/jz501780a. 25400877 PMC4226301

[46] Z. Li , L. F. Song , P. Li , and K. M. Merz , “Systematic Parametrization of Divalent Metal Ions for the OPC3, OPC, TIP3P‐FB, and TIP4P‐FB Water Models,” Journal of Chemical Theory and Computation 16, no. 7 (2020): 4429–4442, 10.1021/acs.jctc.0c00194. 32510956 PMC8173364

[47] D. A. Case , H. M. Aktulga , K. Belfon , et al., Amber 2023 Reference Manual (University of California, 2023).

[48] F. Duarte , P. Bauer , A. Barrozo , et al., “Force Field Independent Metal Parameters Using a Nonbonded Dummy Model,” Journal of Physical Chemistry B 118, no. 16 (2014): 4351–4362, 10.1021/jp501737x. 24670003 PMC4180081

[49] Z. Li , L. F. Song , P. Li , and K. M. Merz , “Parametrization of Trivalent and Tetravalent Metal Ions for the OPC3, OPC, TIP3P‐FB, and TIP4P‐FB Water Models,” Journal of Chemical Theory and Computation 17, no. 4 (2021): 2342–2354, 10.1021/acs.jctc.0c01320. 33793233 PMC8173366

[50] L. X. Peterson , A. Roy , C. Christoffer , G. Terashi , and D. Kihara , “Modeling Disordered Protein Interactions From Biophysical Principles,” PLoS Computational Biology 13, no. 4 (2017): 1–28, 10.1371/journal.pcbi.1005485. PMC5402988 28394890

[51] R. Evans , S. Ramisetty , P. Kulkarni , and K. Weninger , “Illuminating Intrinsically Disordered Proteins With Integrative Structural Biology,” Biomolecules 13, no. 1 (2023): 124, 10.3390/biom13010124. 36671509 PMC9856150

[52] K. M. Ruff and R. V. Pappu , “AlphaFold and Implications for Intrinsically Disordered Proteins,” Journal of Molecular Biology 433, no. 20 (2021): 167208, 10.1016/j.jmb.2021.167208. 34418423

[53] M. van Kempen , S. S. Kim , C. Tumescheit , et al., “Fast and Accurate Protein Structure Search With Foldseek,” Nature Biotechnology 42, no. 2 (2024): 243–246, 10.1038/s41587-023-01773-0. PMC10869269 37156916

[54] R. K. Gundampati , R. Chikati , M. Kumari , et al., “Protein‐Protein Docking on Molecular Models of Aspergillus Niger RNase and Human Actin: Novel Target for Anticancer Therapeutics,” Journal of Molecular Modeling 18, no. 2 (2012): 653–662, 10.1007/s00894-011-1078-4. 21562828

[55] A. Bruley , J. P. Mornon , E. Duprat , and I. Callebaut , “Digging Into the 3D Structure Predictions of AlphaFold2 With Low Confidence: Disorder and Beyond,” Biomolecules 12, no. 10 (2022): 1467, 10.3390/biom12101467. 36291675 PMC9599455

[56] A. Krężel and W. Maret , “The Functions of Metamorphic Metallothioneins in Zinc and Copper Metabolism,” International Journal of Molecular Sciences 18, no. 6 (2017): 1–20, 10.3390/ijms18061237. PMC5486060 28598392

[57] B. Zhao , S. Ghadermarzi , and L. Kurgan , “Comparative Evaluation of AlphaFold2 and Disorder Predictors for Prediction of Intrinsic Disorder, Disorder Content and Fully Disordered Proteins,” Computational and Structural Biotechnology Journal 21 (2023): 3248–3258, 10.1016/j.csbj.2023.06.001. 38213902 PMC10782001

[58] C. J. Wilson , W. Y. Choy , and M. Karttunen , “AlphaFold2: A Role for Disordered Protein/Region Prediction?,” International Journal of Molecular Sciences 23, no. 9 (2022): 1–14, 10.3390/ijms23094591. PMC9104326 35562983

[59] C. A. Blindauer , M. T. Razi , D. J. Campopiano , and P. J. Sadler , “Histidine Ligands in Bacterial Metallothionein Enhance Cluster Stability,” Journal of Biological Inorganic Chemistry 12, no. 3 (2007): 393–405, 10.1007/s00775-006-0196-4. 17203314

[60] M. C. Carpenter , A. Shami Shah , S. Desilva , et al., “Thermodynamics of pb(II) and Zn(II) Binding to MT‐3, a Neurologically Important Metallothionein,” Metallomics 8, no. 6 (2016): 605–617, 10.1039/c5mt00209e. 26757944

[61] M. Remelli , V. M. Nurchi , J. I. Lachowicz , S. Medici , M. A. Zoroddu , and M. Peana , “Competition Between Cd(II) and Other Divalent Transition Metal Ions During Complex Formation With Amino Acids, Peptides, and Chelating Agents,” Coordination Chemistry Reviews 327‐328 (2016): 55–69, 10.1016/j.ccr.2016.07.004.

[62] H. Sigel , R. Griesser , and D. B. McCormick , “On the Structure of Manganese (II)‐ and Copper (II)‐Histidine Complexes,” Archives of Biochemistry and Biophysics 134, no. 1 (1969): 217–227, 10.1016/0003-9861(69)90269-0. 5345587

[63] L. Zhou , S. Li , Y. Su , X. Yi , A. Zheng , and F. Deng , “Interaction Between Histidine and Zn(II) Metal Ions Over a Wide pH as Revealed by Solid‐State NMR Spectroscopy and DFT Calculations,” Journal of Physical Chemistry B 117, no. 30 (2013): 8954–8965, 10.1021/jp4041937. 23841698

[64] A. Grossfield , P. N. Patrone , D. R. Roe , A. J. Schultz , D. Siderius , and D. M. Zuckerman , “Best Practices for Quantification of Uncertainty and Sampling Quality in Molecular Simulations [Article v1.0],” Living Journal of Computational Molecular Science 1, no. 1 (2019): 1–24, 10.33011/livecoms.1.1.5067. PMC6286151 30533602

[65] A. Ganne , M. Balasubramaniam , S. Ayyadevara , and R. J. Shmookler Reis , “Machine‐Learning Analysis of Intrinsically Disordered Proteins Identifies Key Factors That Contribute to Neurodegeneration‐Related Aggregation,” Frontiers in Aging Neuroscience 14 (2022): 1–14, 10.3389/fnagi.2022.938117. PMC9382113 35992603

[66] J. S. Scheller , G. W. Irvine , D. L. Wong , A. Hartwig , and M. J. Stillman , “Stepwise Copper(i) Binding to Metallothionein a Mixed Cooperative and Non‐Cooperative Mechanism for All 20 Copper Ions,” Metallomics 9, no. 5 (2017): 447–462, 10.1039/c7mt00041c. 28466911

[67] L. R. Fernandez , G. Vandenbussche , N. Roosens , C. Govaerts , E. Goormaghtigh , and N. Verbruggen , “Metal Binding Properties and Structure of a Type III Metallothionein From the Metal Hyperaccumulator Plant *Noccaea caerulescens* ,” Biochimica et Biophysica Acta—Proteins and Proteomics 1824, no. 9 (2012): 1016–1023, 10.1016/j.bbapap.2012.05.010. 22668884

[68] M. J. Stillman , W. Cai , and A. J. Zelazowski , “Cadmium Binding to Metallothioneins. Domain Specificity in Reactions of α and β Fragments, Apometallothionein, and Zinc Metallothionein With Cd^2+^ ,” Journal of Biological Chemistry 262, no. 10 (1987): 4538–4548, 10.1016/s0021-9258(18)61226-8. 3558354

[69] R. Morita , Y. Shigeta , and R. Harada , “Structural Variations of Metallothionein With or Without Zinc Ions Elucidated by All‐Atom Molecular Dynamics Simulations,” Journal of Physical Chemistry B 125, no. 46 (2021): 12712–12717, 10.1021/acs.jpcb.1c07928. 34762438

[70] M. Serra‐Batiste , N. Cols , L. A. Alcaraz , A. Donaire , P. González‐Duarte , and M. Vašák , “The Metal‐Binding Properties of the Blue Crab Copper Specific CuMT‐2: A Crustacean Metallothionein With Two Cysteine Triplets,” Journal of Biological Inorganic Chemistry 15, no. 5 (2010): 759–776, 10.1007/s00775-010-0644-z. 20361221

[71] G. Yuan , F. Curtolo , Y. Deng , et al., “Highly Dynamic Polynuclear Metal Cluster Revealed in a Single Metallothionein Molecule,” Research 2021 (2021): 1–11, 10.34133/2021/9756945. PMC8299258 34368766

[72] G. W. Irvine , N. Korkola , and M. J. Stillman , “Isolated Domains of Recombinant Human Apo‐Metallothionein 1A Are Folded at Neutral pH: A Denaturant and Heat‐Induced Unfolding Study Using ESI‐MS,” Bioscience Reports 38, no. 4 (2018): 1–10, 10.1042/BSR20180592. PMC6050192 29858425

[73] N. C. Korkola and M. J. Stillman , “Human Apo‐Metallothionein 1a Is Not a Random Coil: Evidence From Guanidinium Chloride, High Temperature, and Acidic pH Unfolding Studies,” Biochimica et Biophysica Acta 1872, no. 4 (2024): 141010, 10.1016/j.bbapap.2024.141010. 38490456

[74] S. H. Chen , L. Chen , and D. H. Russell , “Metal‐Induced Conformational Changes of Human Metallothionein‐2A: A Combined Theoretical and Experimental Study of Metal‐Free and Partially Metalated Intermediates,” Journal of the American Chemical Society 136, no. 26 (2014): 9499–9508, 10.1021/ja5047878. 24918957

[75] D. H. Petering , J. Zhu , S. Krezoski , et al., “Apo‐Metallothionein Emerging as a Major Player in the Cellular Activities of Metallothionein,” Experimental Biology and Medicine 231, no. 9 (2006): 1528–1534, 10.1177/153537020623100912. 17018876

[76] K. E. Rigby Duncan and M. J. Stillman , “Metal‐Dependent Protein Folding: Metallation of Metallothionein,” Journal of Inorganic Biochemistry 100, no. 12 (2006): 2101–2107, 10.1016/j.jinorgbio.2006.09.005. 17055583

[77] E. A. Peroza , R. Schmucki , P. Güntert , E. Freisinger , and O. Zerbe , “The βE‐Domain of Wheat Ec‐1 Metallothionein: A Metal‐Binding Domain With a Distinctive Structure,” Journal of Molecular Biology 387, no. 1 (2009): 207–218, 10.1016/j.jmb.2009.01.035. 19361445

[78] M. Perinelli , M. Tegoni , and E. Freisinger , “Different Behavior of the Histidine Residue Toward Cadmium and Zinc in a Cadmium‐Specific Metallothionein From an Aquatic Fungus,” Inorganic Chemistry 59, no. 23 (2020): 16988–16997, 10.1021/acs.inorgchem.0c02171. 33205965

[79] B. Gold , H. Deng , R. Bryk , et al., “Identification of a Copper‐Binding Metallothionein in Pathogenic Mycobacteria,” Nature Chemical Biology 4, no. 10 (2008): 609–616, 10.1038/nchembio.109. 18724363 PMC2749609

[80] Y. He , Z. Jiang , M. Zeng , S. Cao , N. Wu , and X. Liu , “Unraveling Potential Mechanism of Different Metal Ions Effect on Anammox Through Big Data Analysis, Molecular Docking and Molecular Dynamics Simulation,” Journal of Environmental Management 352 (2024): 120092, 10.1016/j.jenvman.2024.120092. 38232596

[81] K. Mitusińska , T. Skalski , and A. Góra , “Simple Selection Procedure to Distinguish Between Static and Flexible Loops,” International Journal of Molecular Sciences 21, no. 7 (2020): 2293, 10.3390/ijms21072293. 32225102 PMC7177474

[82] T. B. J. Pinter , G. W. Irvine , and M. J. Stillman , “Domain Selection in Metallothionein 1A: Affinity‐Controlled Mechanisms of Zinc Binding and Cadmium Exchange,” Biochemistry 54, no. 32 (2015): 5006–5016, 10.1021/acs.biochem.5b00452. 26167879

[83] H. Wang , Q. Zhang , B. Cai , et al., “Solution Structure and Dynamics of Human Metallothionein‐3 (MT‐3),” FEBS Letters 580, no. 3 (2006): 795–800, 10.1016/j.febslet.2005.12.099. 16413543

[84] A. Drozd , D. Wojewska , M. D. Peris‐Díaz , P. Jakimowicz , and A. Krȩzel , “Crosstalk of the Structural and Zinc Buffering Properties of Mammalian Metallothionein‐2,” Metallomics 10, no. 4 (2018): 595–613, 10.1039/c7mt00332c. 29561927

[85] J. S. Scheller , G. W. Irvine , and M. J. Stillman , “Unravelling the Mechanistic Details of Metal Binding to Mammalian Metallothioneins From Stoichiometric, Kinetic, and Binding Affinity Data,” Dalton Transactions 47, no. 11 (2018): 3613–3637, 10.1039/c7dt03319b. 29431781

[86] F. A. Cotton and G. Wilkinson , Advanced Inorganic Chemistry: A Comprehensive Text, 5th ed. (Wiley‐lnterscience, 1980).

[87] H. Irving and R. J. P. Williams , “Order of Stability of Metal Complexes,” Nature 162, no. 4123 (1948): 746–747, 10.1038/162746a0.

[88] M. Belcastro , T. Marino , N. Russo , and M. Toscano , “Interaction of Cysteine With cu 2+ and Group IIb (Zn2+, Cd2+, Hg2+) Metal Cations: A Theoretical Study,” Journal of Mass Spectrometry 40, no. 3 (2005): 300–306, 10.1002/jms.755. 15685654

[89] Ò. Palacios and M. Capdevila , “Metallothioneins and Lead,” in Encyclopedia of Metalloproteins, vol. 5 (Springer, 2013), 1383–1386, 10.1007/978-1-4614-1533-6_319.

[90] D. L. Wong , M. E. Merrifield‐MacRae , and M. J. Stillman , “Lead(II) Binding in Metallothioneins,” Lead: Its Effects on Environment and Health 17, no. 2 (2017): 241–269, 10.1515/9783110434330-009. 28731302

[91] Y. He , M. Liu , N. Darabedian , et al., “PH‐Dependent Coordination of Pb2+ to Metallothionein2: Structures and Insight Into Lead Detoxification,” Inorganic Chemistry 53, no. 6 (2014): 2822–2830, 10.1021/ic402452s. 24559479 PMC3993925

[92] Ò. Palacios , À. Leiva‐Presa , S. Atrian , and R. Lobinski , “A Study of the pb(II) Binding to Recombinant Mouse Zn7‐Metallothionein 1 and Its Domains by ESI TOF MS,” Talanta 72, no. 2 (2007): 480–488, 10.1016/j.talanta.2006.11.009. 19071644

[93] A. A. Bhopatkar , V. N. Uversky , and V. Rangachari , Disorder and Cysteines in Proteins: A Design for Orchestration of Conformational See‐Saw and Modulatory Functions, vol. 174, 1st ed. (Elsevier Inc, 2020), 10.1016/bs.pmbts.2020.06.001. PMC8048112 32828470

[94] H. Kozłowski , W. Bal , M. Dyba , and T. Kowalik‐Jankowska , “Specific Structure–Stability Relations in Metallopeptides,” Coordination Chemistry Reviews 184, no. 1 (1999): 319–346, 10.1016/S0010-8545(98)00261-6.

[95] K. Garstka , V. Dzyhovskyi , J. Wątły , et al., “CH vs. HC—Promiscuous Metal Sponges in Antimicrobial Peptides and Metallophores,” Molecules 28, no. 10 (2023): 3985, 10.3390/molecules28103985. 37241727 PMC10221980

[96] A. Muñoz , D. H. Petering , and C. F. Shaw , “Structure—Reactivity Relationships Among Metallothionein Three‐Metal Domains: Role of Non‐Cysteine Amino Acid Residues in Lobster Metallothionein and Human Metallothionein‐3,” Inorganic Chemistry 39, no. 26 (2000): 6114–6123, 10.1021/ic000485s. 11188527

[97] A. K. Singh , A. Pomorski , S. Wu , M. D. Peris‐Díaz , H. Czepczyńska‐Krȩzel , and A. Krȩzel , “The Connection of α‐And β‐Domains in Mammalian Metallothionein‐2 Differentiates Zn(II) Binding Affinities, Affects Folding, and Determines Zinc Buffering Properties,” Metallomics 15, no. 6 (2023): 1–20, 10.1093/mtomcs/mfad029. PMC10243857 37147085

[98] Ò. Palacios , A. Pagani , S. Pérez‐Rafael , et al., “Shaping Mechanisms of Metal Specificity in a Family of Metazoan Metallothioneins: Evolutionary Differentiation of Mollusc Metallothioneins,” BMC Biology 9, no. 1 (2011): 4, 10.1186/1741-7007-9-4. 21255385 PMC3033865

[99] K. P. Neupane and V. L. Pecoraro , “Pb‐207 NMR Spectroscopy Reveals That Pb(II) Coordinates With Glutathione (GSH) and Tris Cysteine Zinc Finger Proteins in a PbS3 Coordination Environment,” Journal of Inorganic Biochemistry 105, no. 8 (2011): 1030–1034, 10.1016/j.jinorgbio.2011.04.010. 21625408 PMC3101507

[100] H. C. Gonick , “Lead‐Binding Proteins: A Review,” Journal of Toxicology 2011 (2011): 1–10, 10.1155/2011/686050. PMC3175699 21941540

[101] L. Mollica , L. M. Bessa , X. Hanoulle , M. R. Jensen , M. Blackledge , and R. Schneider , “Binding Mechanisms of Intrinsically Disordered Proteins: Theory, Simulation, and Experiment,” Frontiers in Molecular Biosciences 3 (2016): 1–18, 10.3389/fmolb.2016.00052. 27668217 PMC5016563

