# Supplementary material for: Metal‐Coordination Specificity and Structural Dynamics of C. elegans Metallothionein I: Insights From 3D Modeling and MD Simulations
Source: Proteins. 2025 Sep 21;94(2):620–32. doi: 10.1002/prot.70054 (PMC12779199; doi:10.1002/prot.70054)
Supplement: Supplementary file 1 — Data S1: Supporting Information. [file PROT-94-620-s001.docx]

**Supplementary material**

Title: **Metal-coordination specificity and structural dynamics of C. elegans metallothionein I:**

**insights from 3D modeling and MD simulations**

Running title: ***C. elegans* MTL-1: Metal coordination and Dynamics**

Nilvea Ramalho de Oliveira^1*^, Andrei Santos Siqueira ^2^, Paulo Sérgio Alves Bueno ^3^, Evonnildo Costa Gonçalves ^2^, Juliano Zanette^1^

1 - Universidade Federal do Rio Grande, Instituto de Ciências Biológicas, Av. Itália, km 8, CEP: 96203-900, Campus Carreiros, Rio Grande – RS, Brazil

2 - Universidade Federal do Pará, Laboratório de Tecnologia Biomolecular, Instituto de Ciências Biológicas, Rua Augusto Corrêa, 01, CEP 66075-110, Campus Universitário do Guamá, Belém, PA, Brazil.

3 - Universidade Estadual de Maringá, Departamento de Bioquímica, Av. Colombo, 5790 Cep: 87020-900, Jd. Universitário, Maringá, Paraná, Brazil.

**Table S1**. Percentage of secondary structure of MTL-1 3D model from AlphaFold and after molecular dynamics (200 ns) in AMBER, shown for duplicate simulations. The secondary structure was determined quantitatively using the DSSP program (Kabsch & Sander, 1983).

| Secondary Structure (%) | MTL-1 3D model  AlphaFold | MTL-1 Apo  (Replica 1) | MTL-1 Apo  (Replica2) |
| --- | --- | --- | --- |
| Bend | 34.7 | 21.3 | 16.0 |
| Turn | 13.3 | 8.0 | 12.0 |
| Alpha Helix (4-12) | 6.7 | 0.0 | 10.7 |
| 3-10 Helix | 4.0 | 13.3 | 4.0 |
| Coil | 41.3 | 57.3 | 57.3 |
|  |  |  |  |
| Total Bend/Turn/Coil | 89.3 | 86.6 | 85.3 |


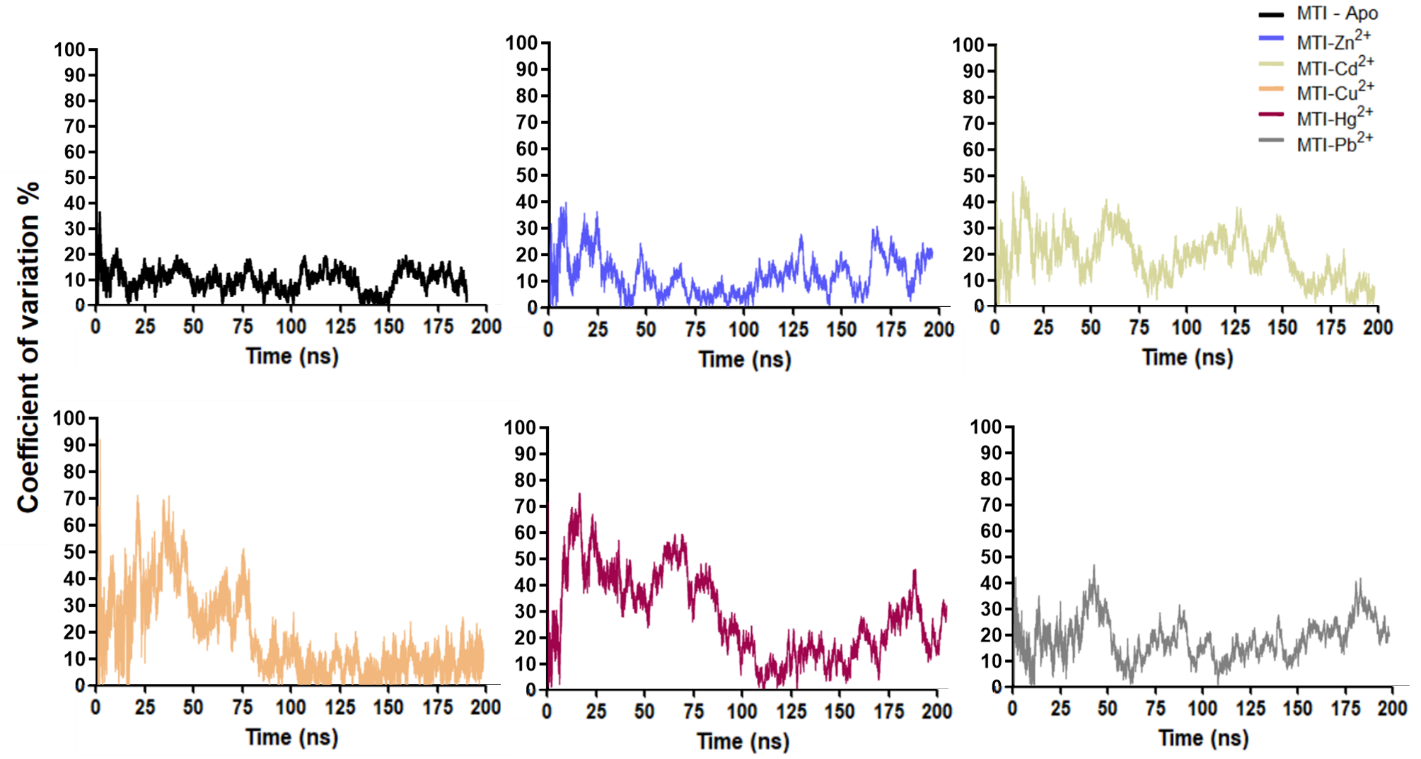


**Figure S1.** Coefficient of variation (%) of the RMSD (root mean square deviation) parameter for triplicate molecular dynamics simulations of all atoms of isoform I of metallothionein from *Caenorhabditis elegans*, in the absence of metal ions (MTL-1 Apo) and with various divalent metal ion coordination.


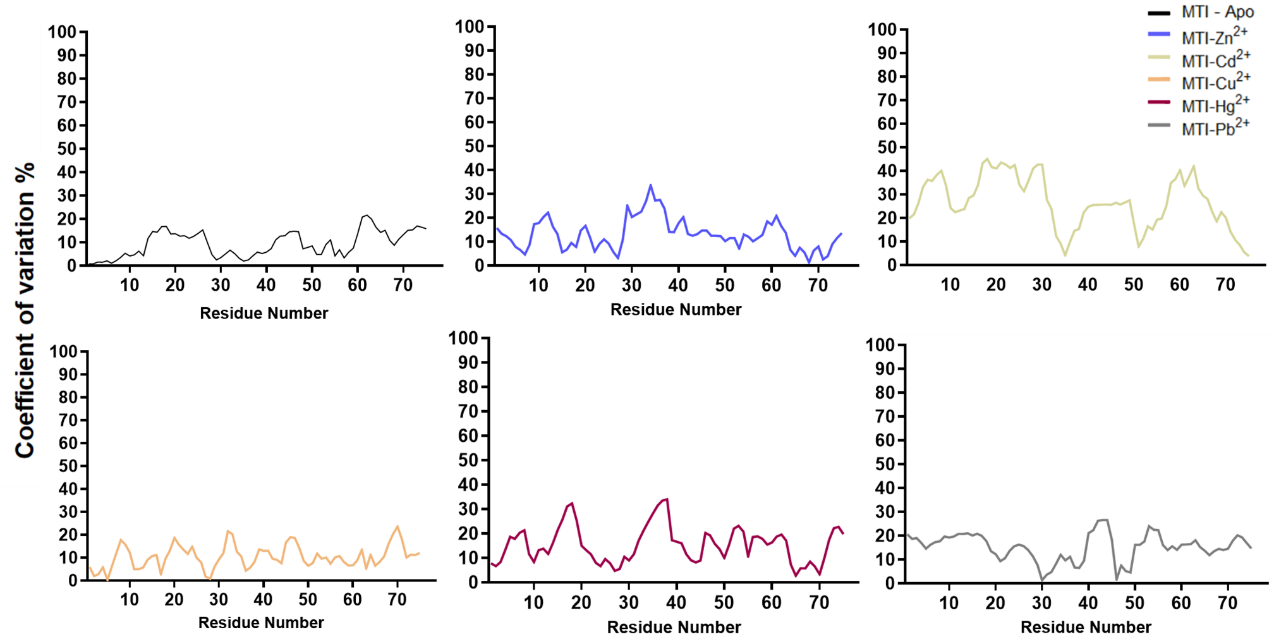


**Figure S2**. Coefficient of variation (%) of the RMSF (root mean square fluctuation) parameter for triplicate molecular dynamics simulations of all atoms of isoform I of metallothionein from *Caenorhabditis elegans*, in the absence of metal ions (MTL-1 Apo) and with various divalent metal ion coordination.


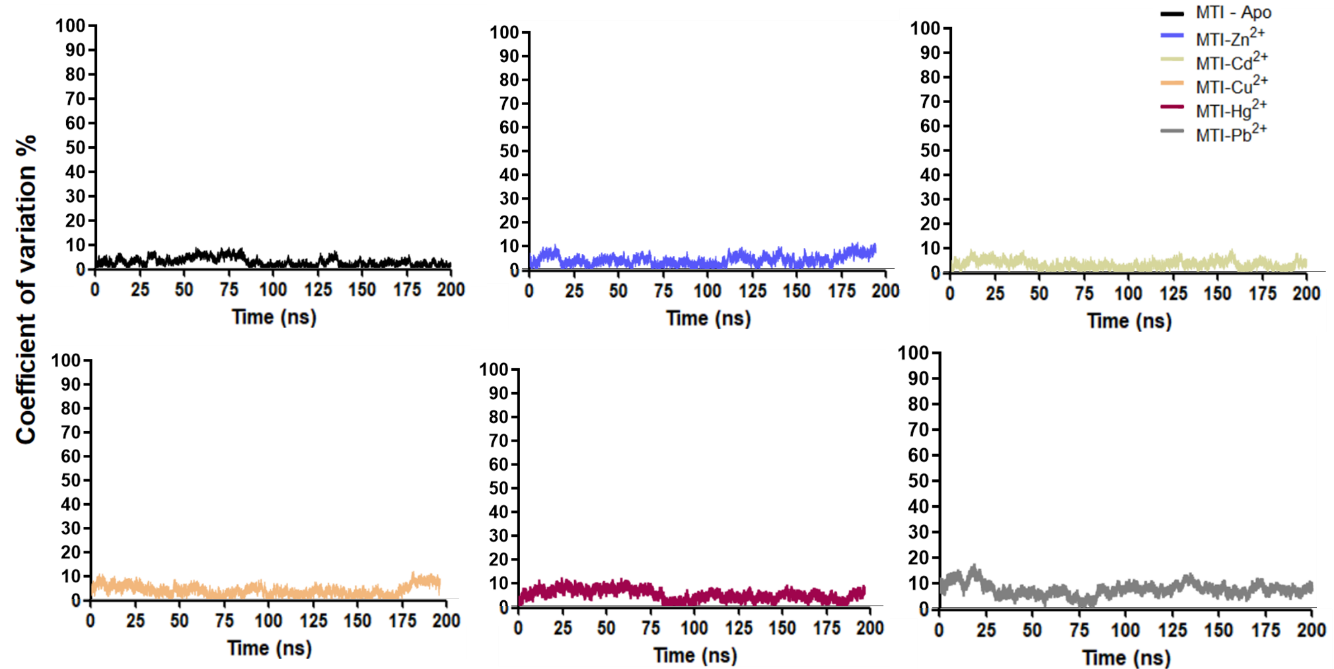


**Figure S3.** Coefficient of variation (%) of the SASA (accessible surface area) parameter for triplicate molecular dynamics simulations of all atoms of isoform I of metallothionein from *Caenorhabditis elegans*, in the absence of metal ions (MTL-1 Apo) and with various divalent metal ion coordination.


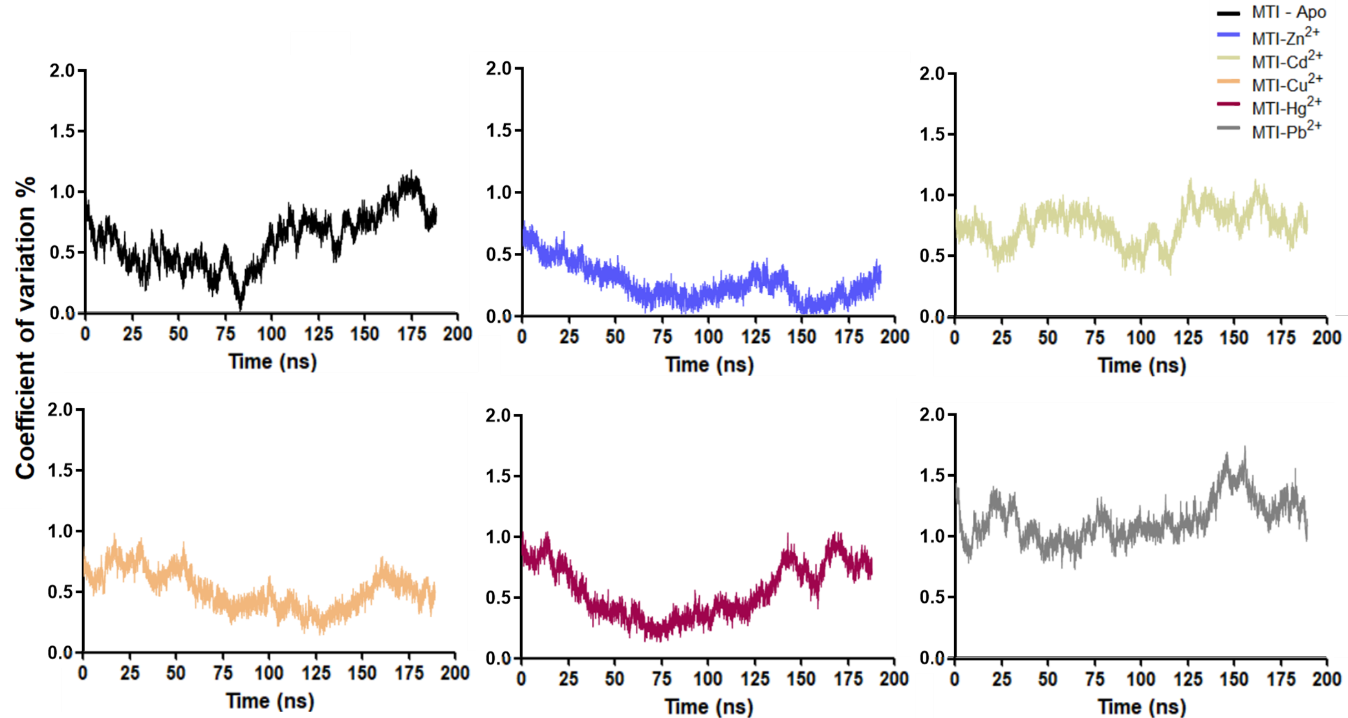


**Figure S4.** Coefficient of variation (%) of radius of gyration (R_g_) parameter for triplicate molecular dynamics simulations of all atoms of isoform I of metallothionein from *Caenorhabditis elegans*, in the absence of metal ions (MTL-1 Apo) and with various divalent metal ion coordination.

| **Réplica 1** | **Réplica 2** | **Réplica 3** |
| --- | --- | --- |
| 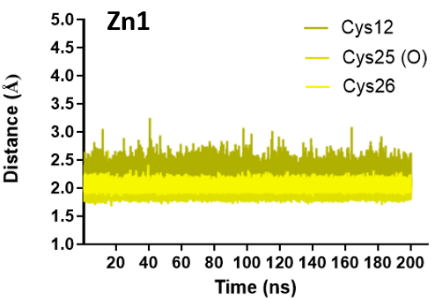 | 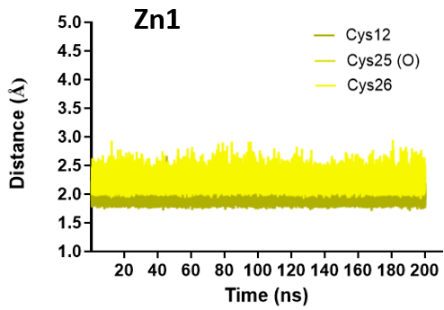 | **Zn1**  **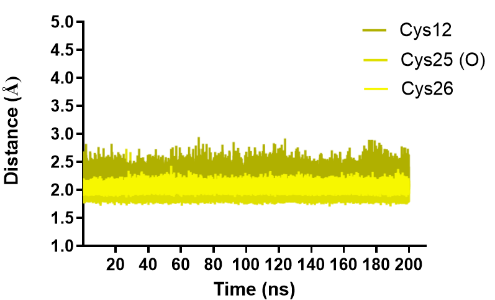** |
| 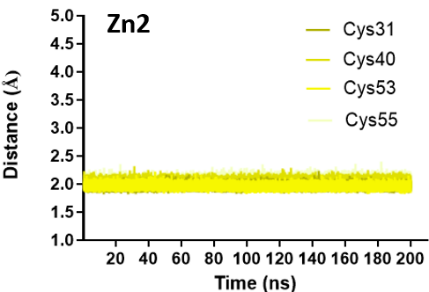 | 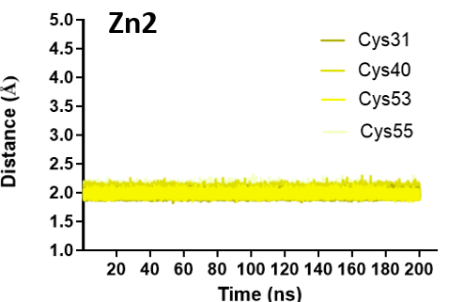 | **Zn2**  **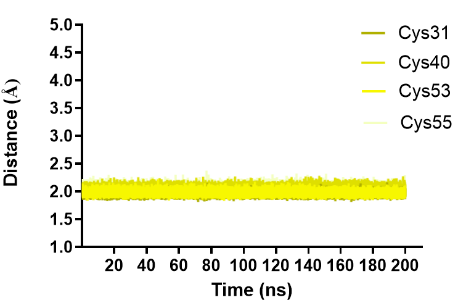** |
| 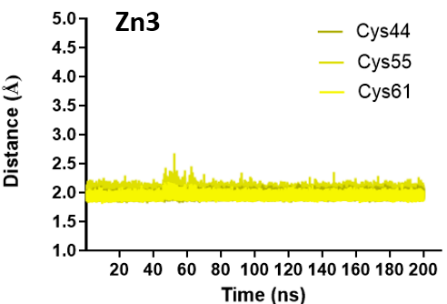 | 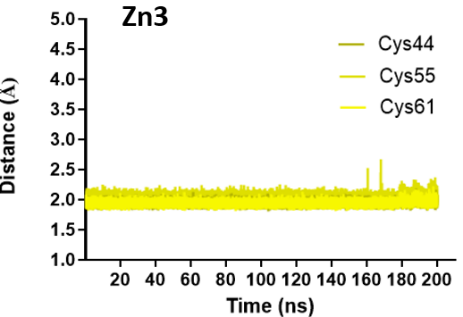 | **Zn3**  **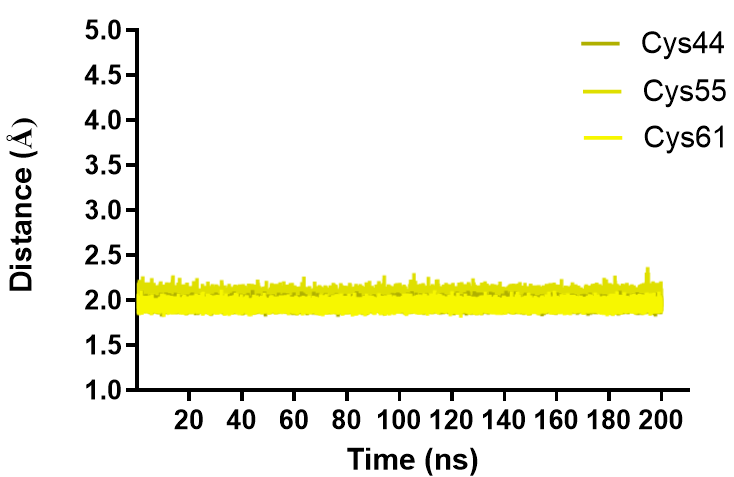** |
| 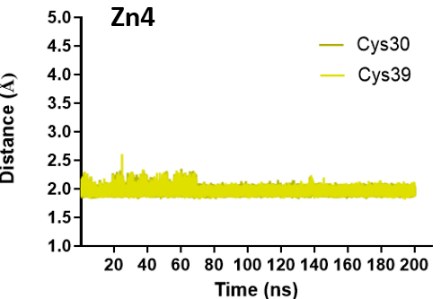 | 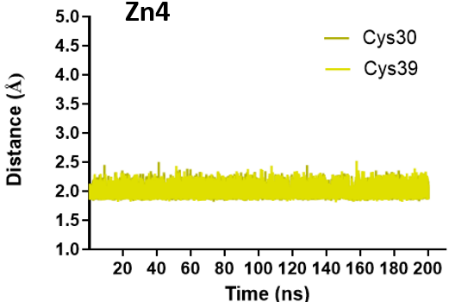 | **Zn4**  **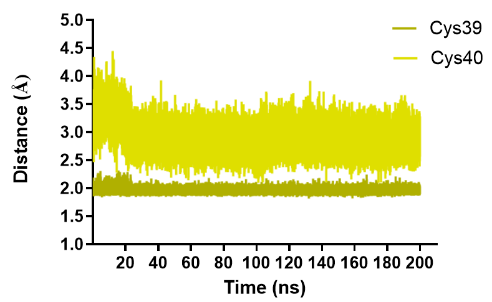** |
| 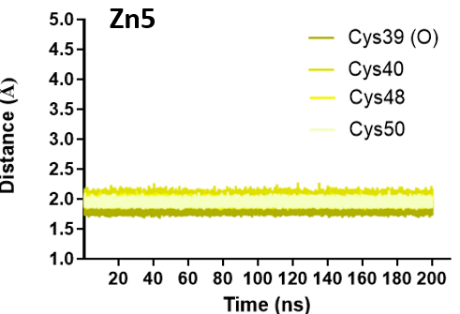 | 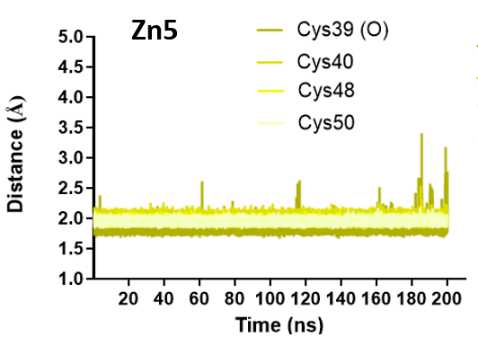 | **Zn5**  **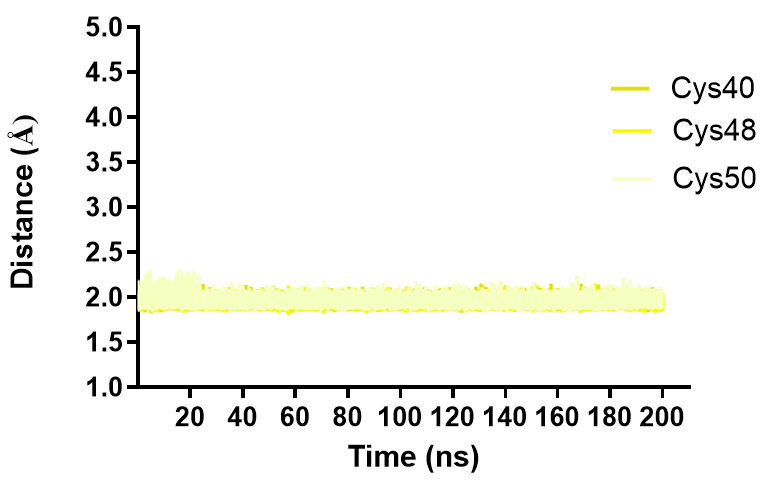** |
| 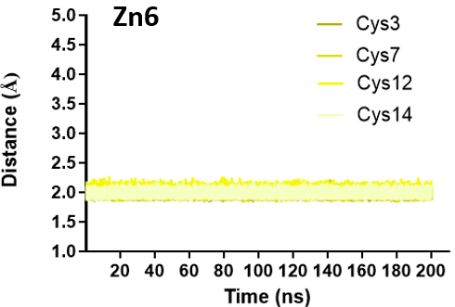 | 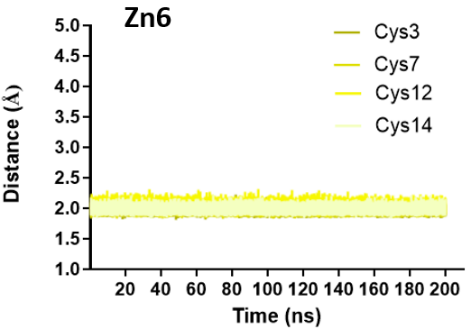 | **Zn6**  **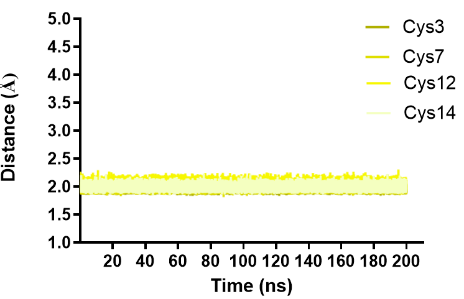** |
| 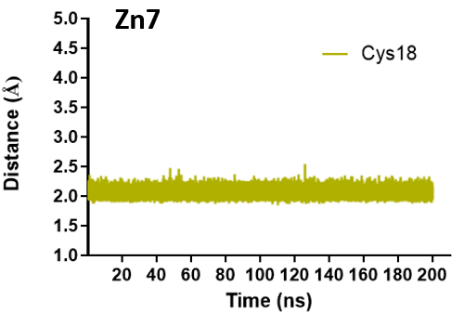 | 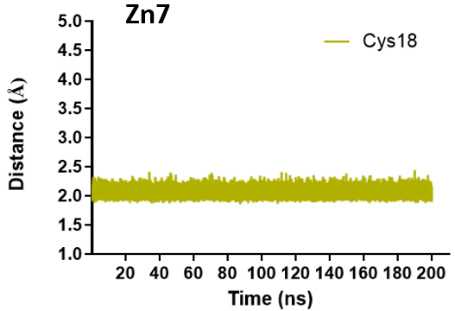 | **Zn7**  **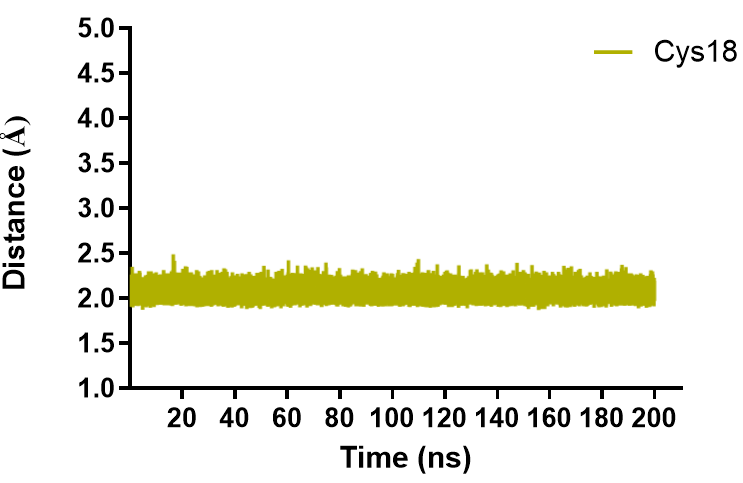** |
| **Figure S5.** Distance in Angstroms > 2.5, between residues and the Zn metal at each ion coordination site (1-7) | | |

| **Replica 1** | **Replica 2** | **Replica 3** |
| --- | --- | --- |
| 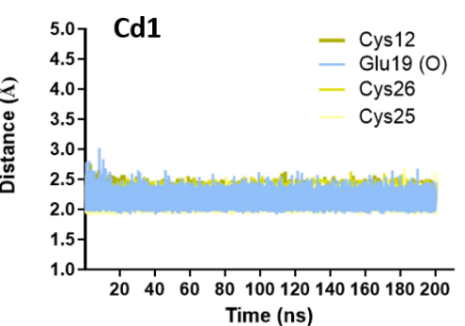 | 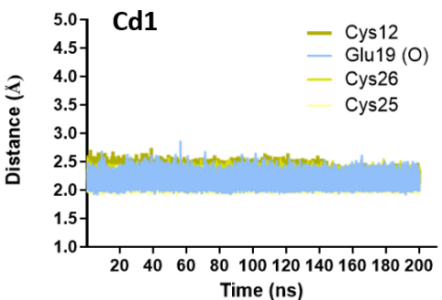 | **Cd1**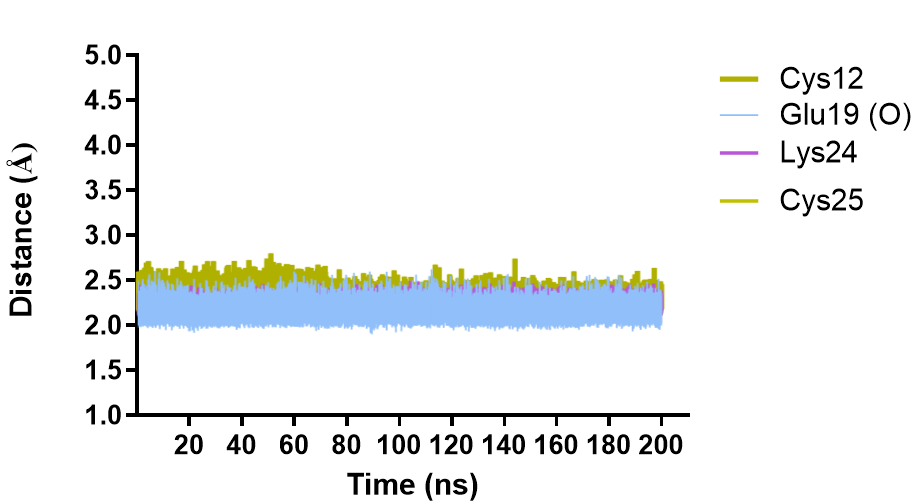 |
| **Cd2**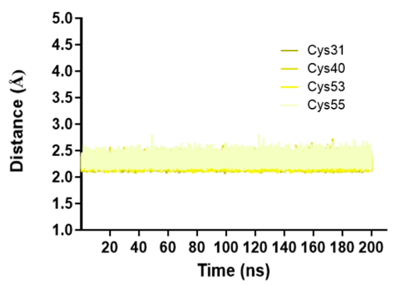 | 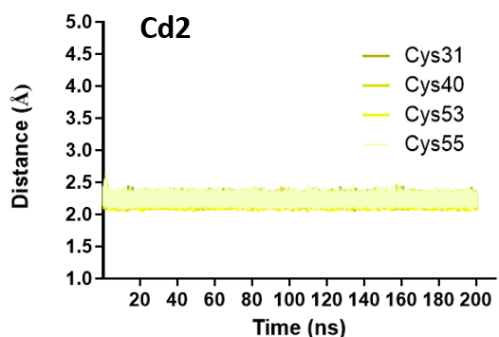 | **Cd2**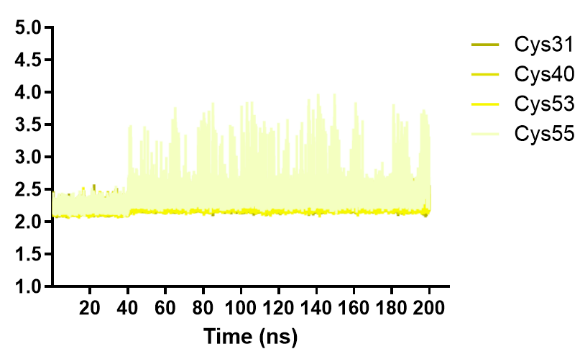 |
| 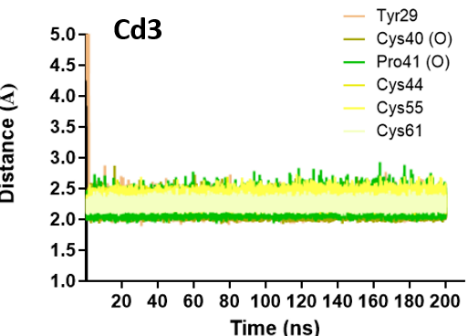 | 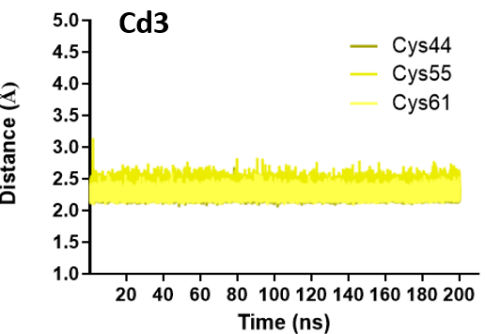 | **Cd3**  **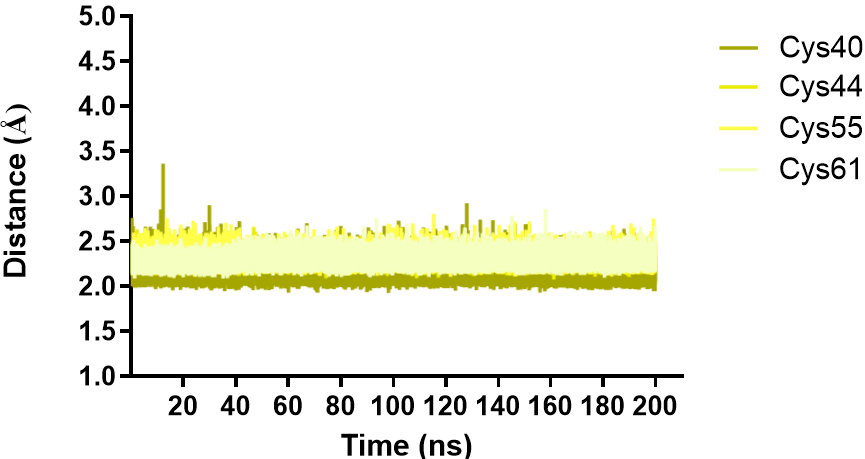** |
| 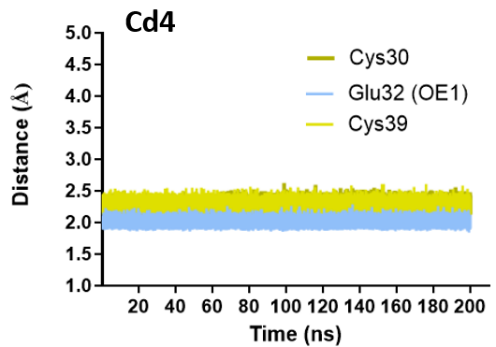 | 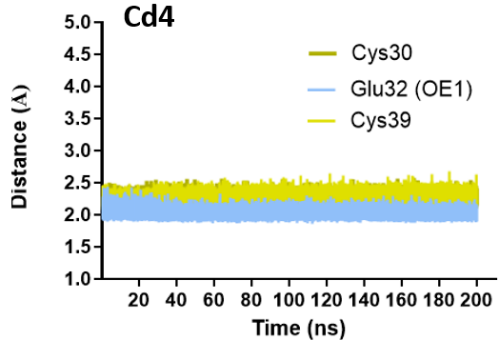 | **Cd4**  **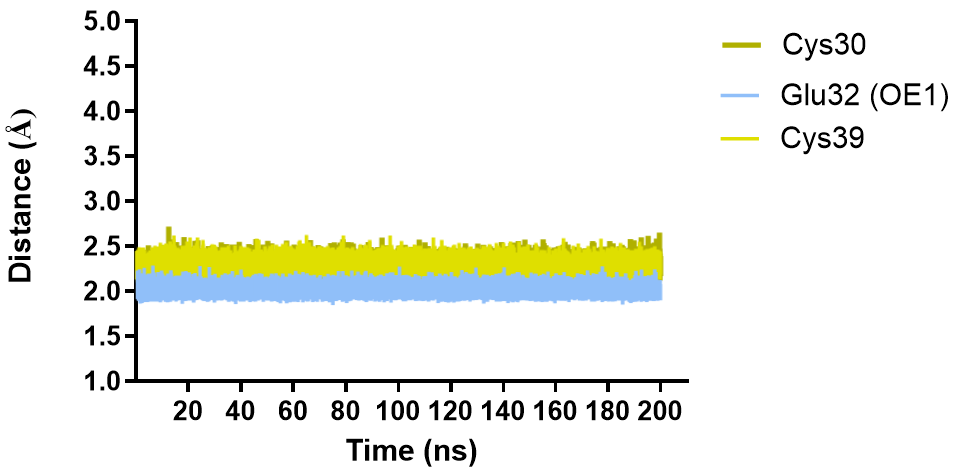** |
| 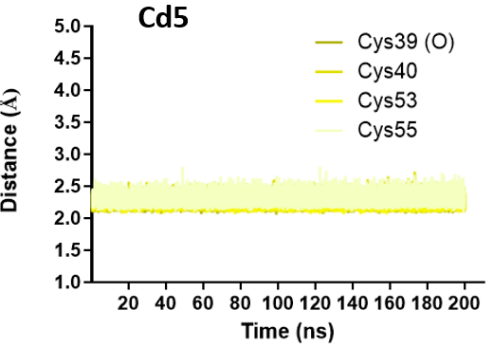 | 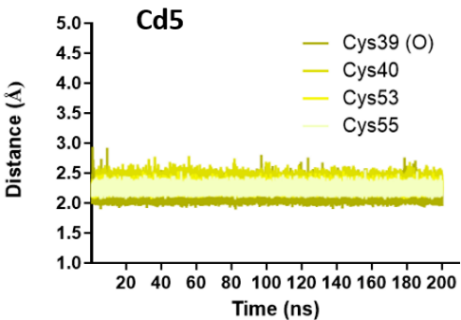 | **Cd5**  **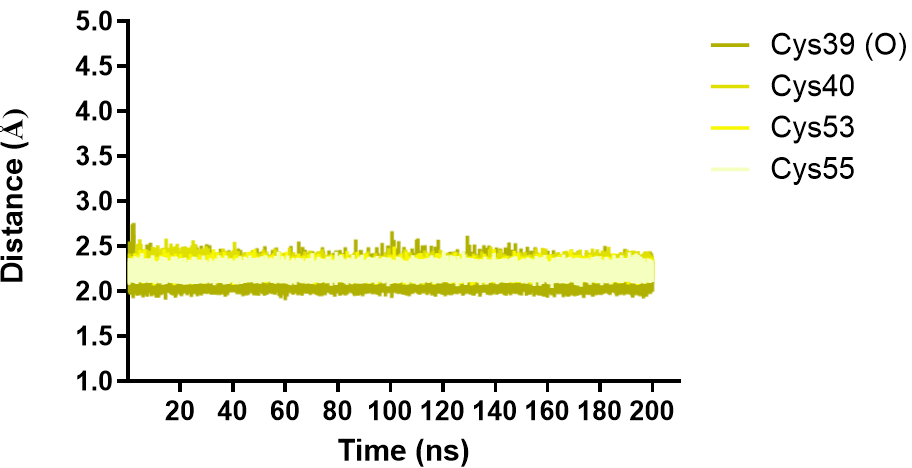** |
| 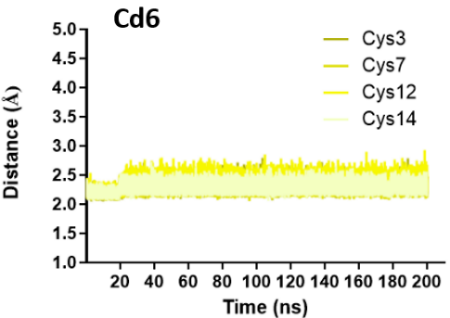 | 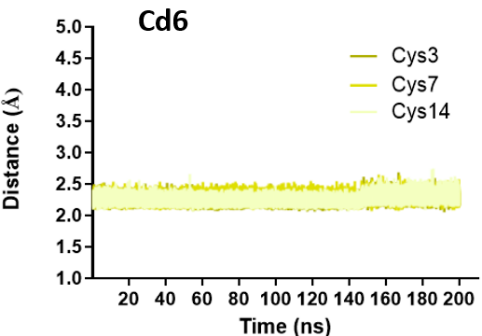 | **Cd6**  **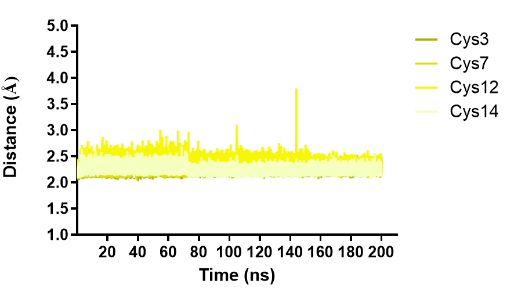** |
| 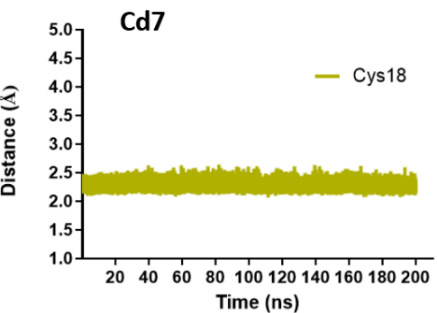 | 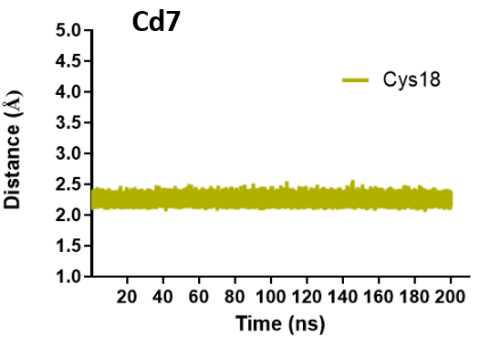 | **Cd7**  **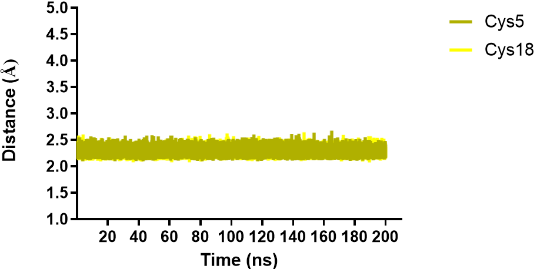** |
| **Figure S6.** Distance in Angstroms > 2.5, between residues and the Cd metal at each ion coordination site (1-7) | | |

| **Replica 1** | **Replica 2** | **Replica 3** |
| --- | --- | --- |
| 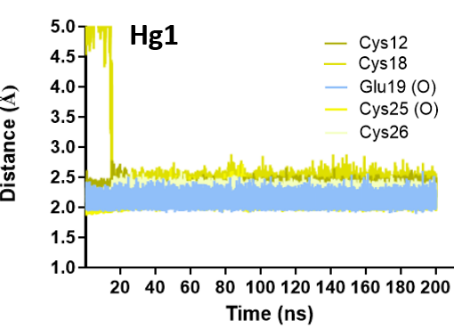 | 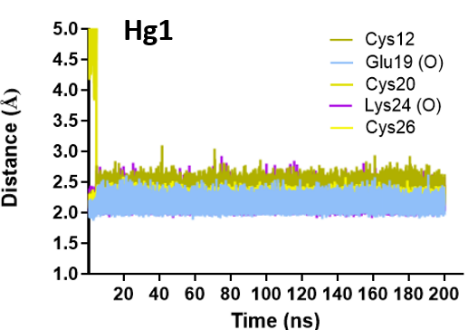 | **Hg1**  **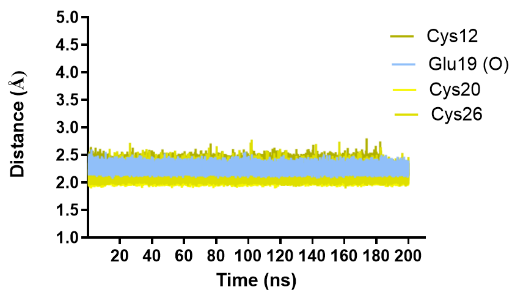** |
| 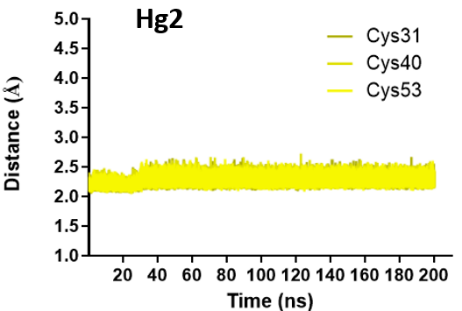 | 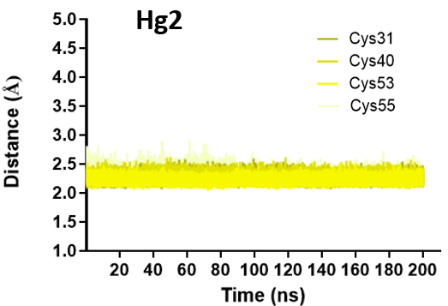 | **Hg2**  **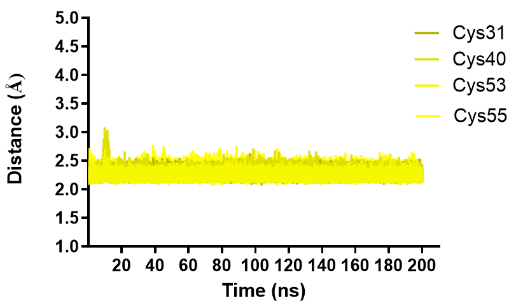** |
| 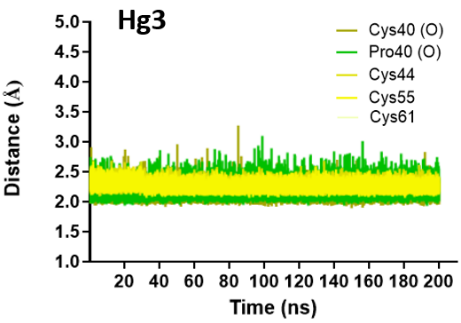 | 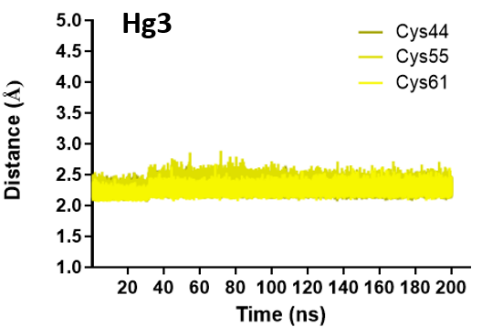 | **Hg3**  **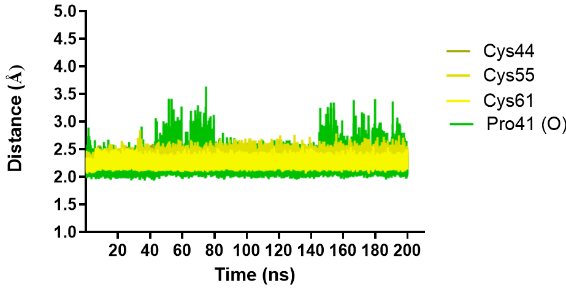** |
| 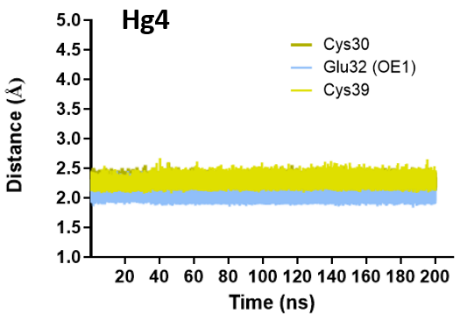 | 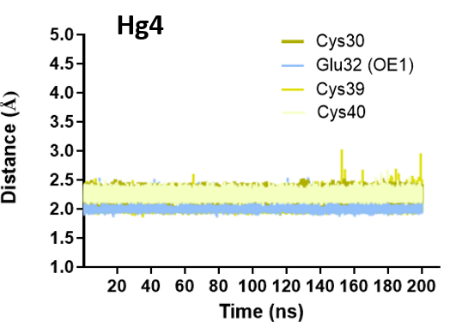 | **Hg4**  **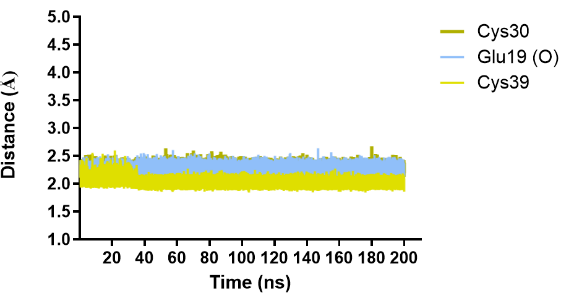** |
| 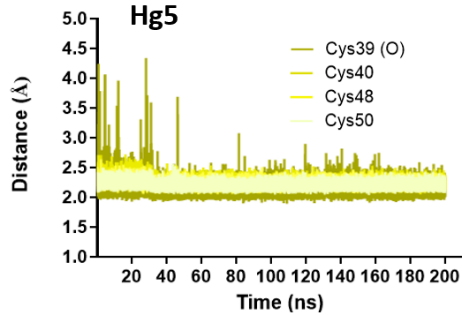 | 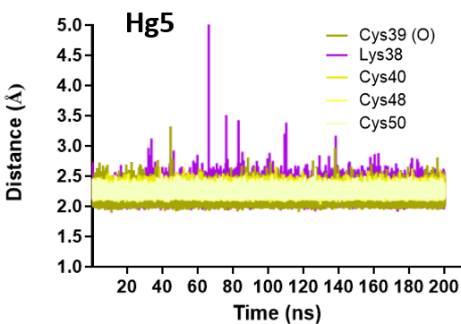 | **Hg5**  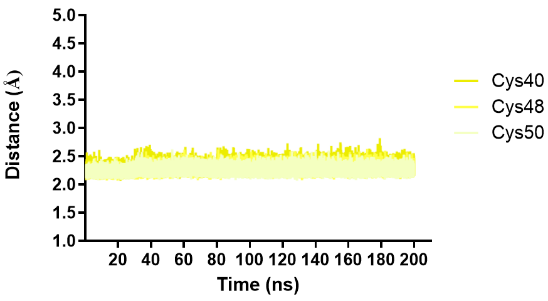 |
| 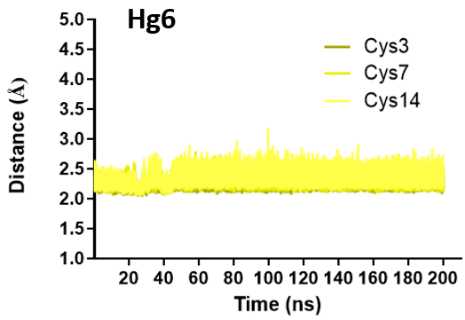 | 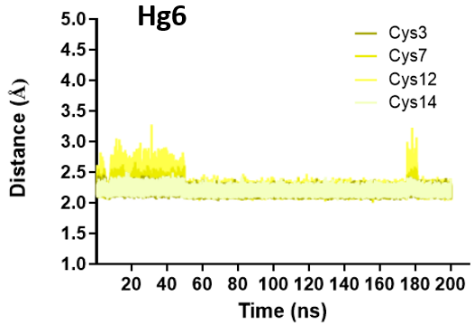 | **Hg6**  **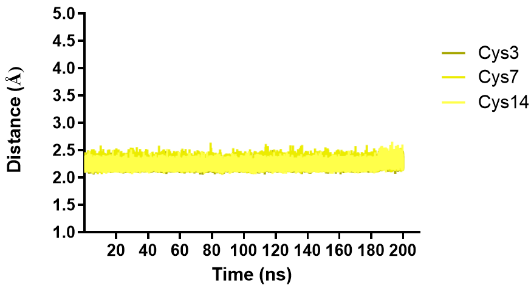** |
| 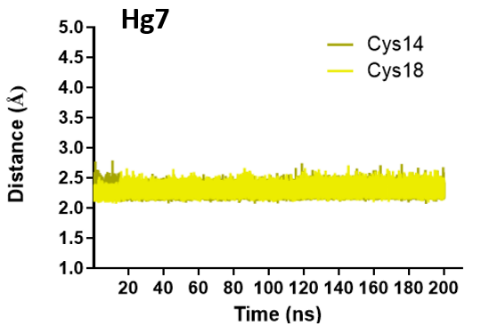 | 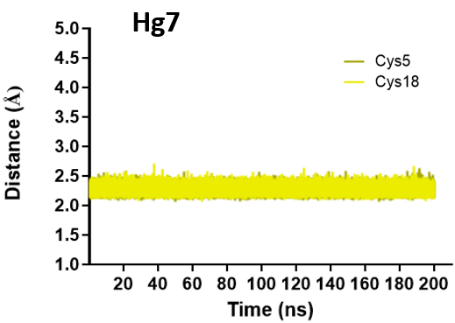 | **Hg7**  **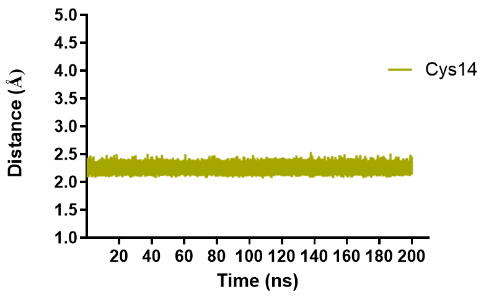** |
| **Figure S7.** Distance in Angstroms > 2.5, between residues and the Hg metal at each ion coordination site (1-7) | | |

| **Replica1** | **Replica2** | **Replica 3** | |
| --- | --- | --- | --- |
| 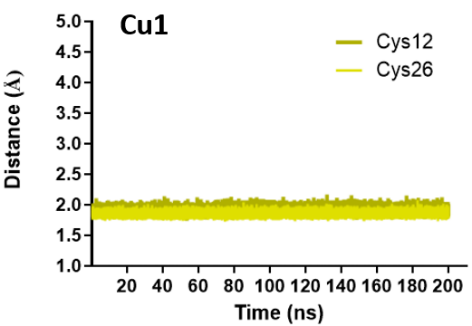 | 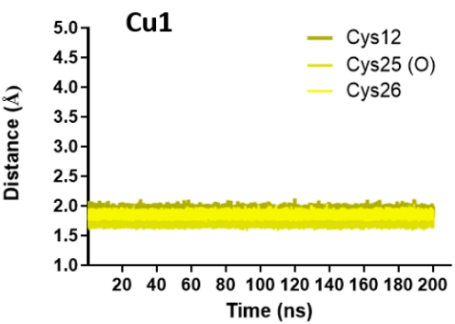 | **Cu1**  **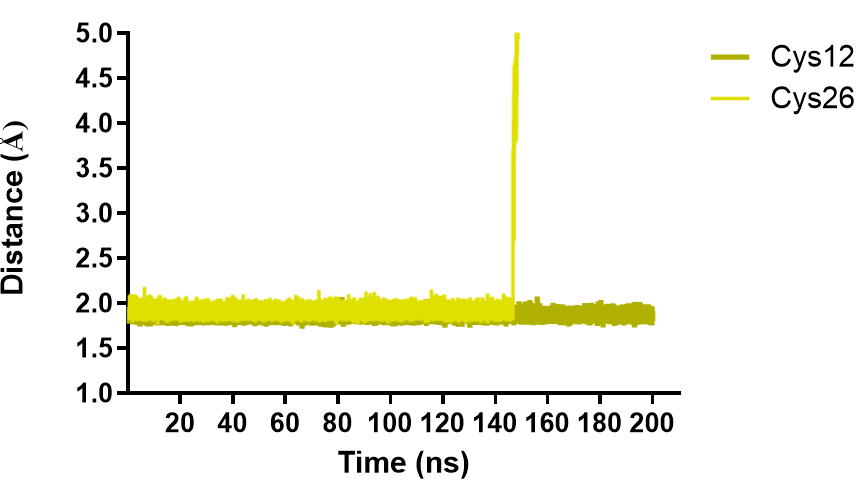** | |
| 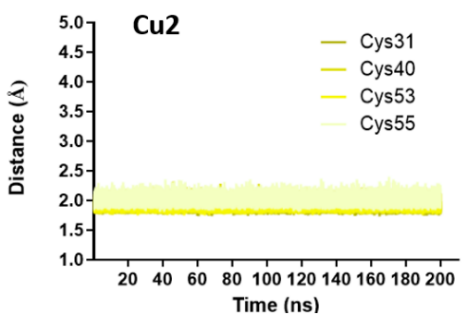 | 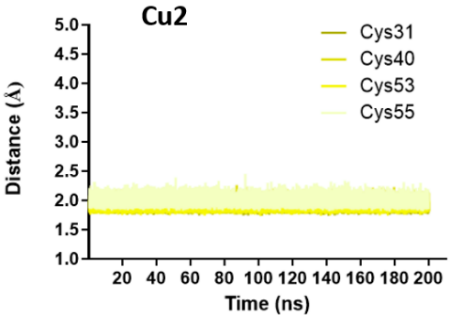 | **Cu2**  **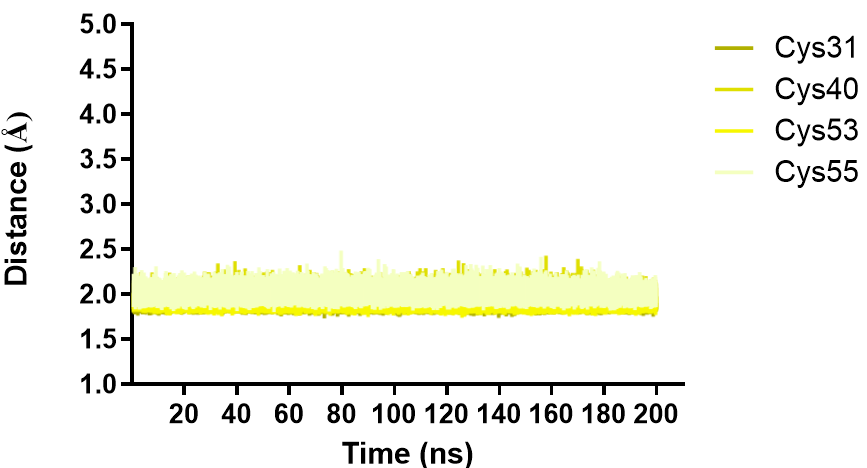** | |
| 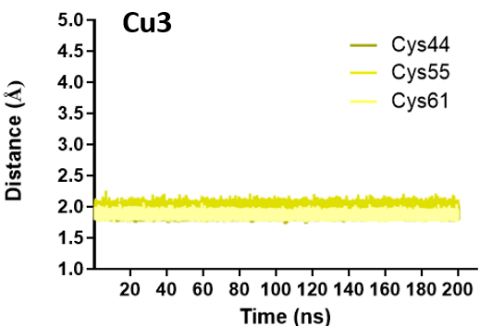 | 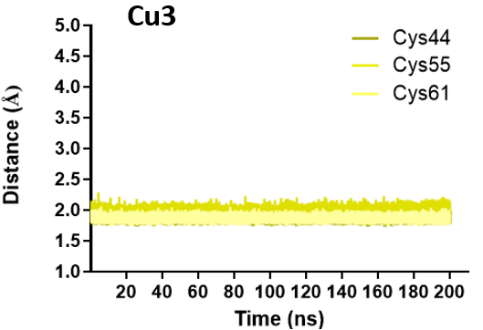 | **Cu3**  **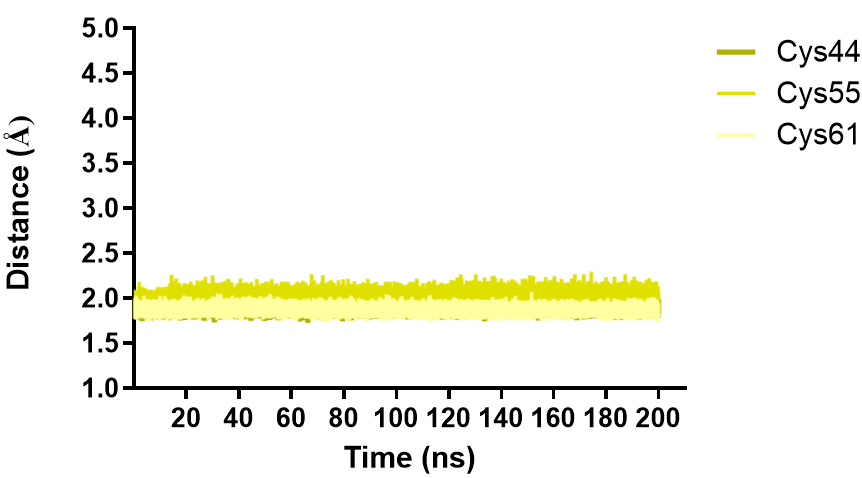** | |
| 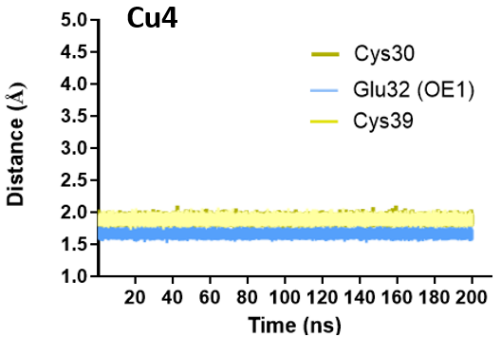 | 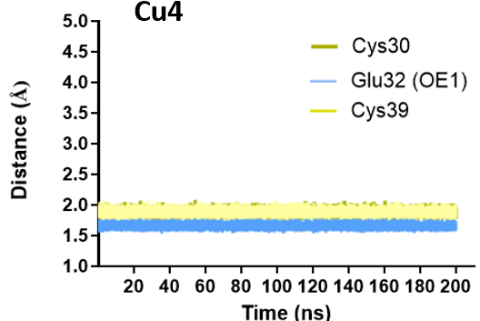 | **Cu4**  **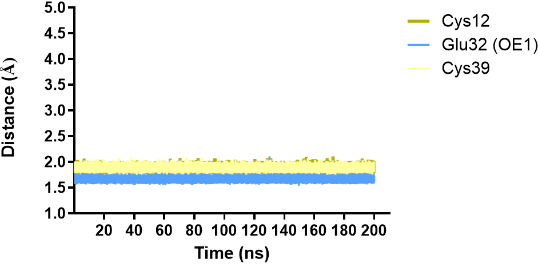** | |
| 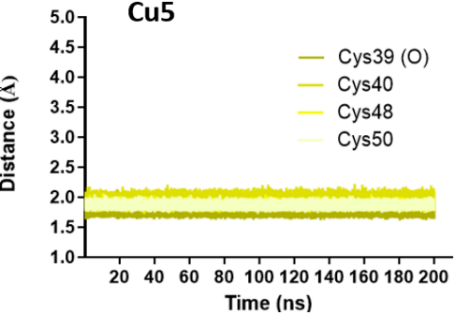 | 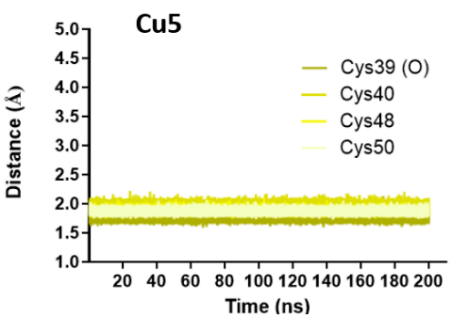 | **Cu5**  **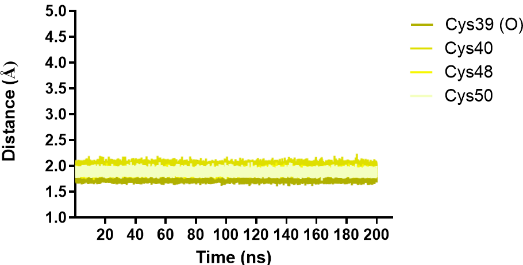** | |
| 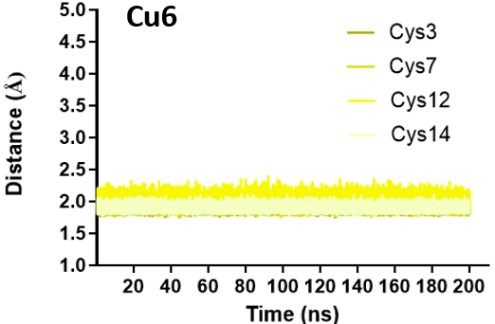 | 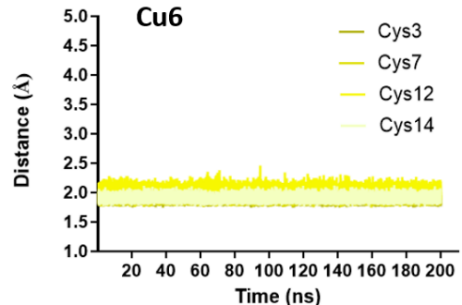 | **Cu6**  **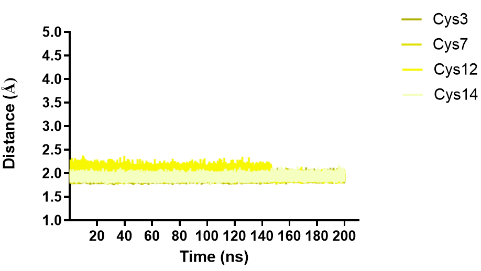** | |
| 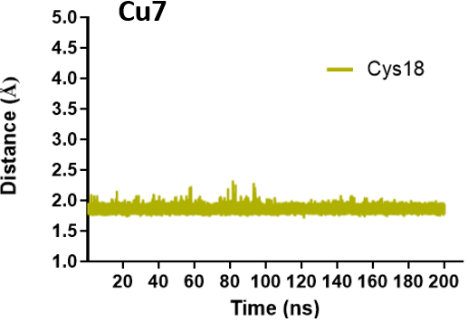 | 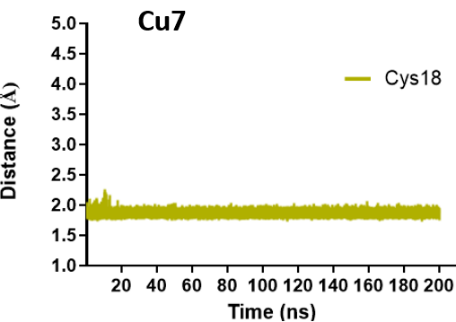 | **Cu7**  **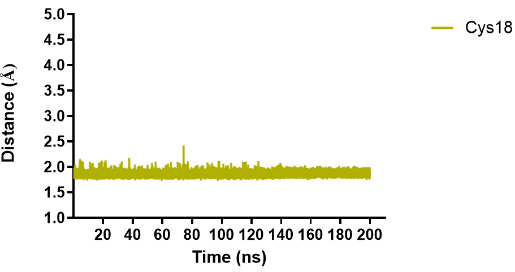** | |
| **Figure S8.** Distance in Angstroms > 2.5, between residues and the Cu metal at each ion coordination site (1-7) | | |  |

| **Replica 1** | **Replica 2** | **Replica 3** |
| --- | --- | --- |
| **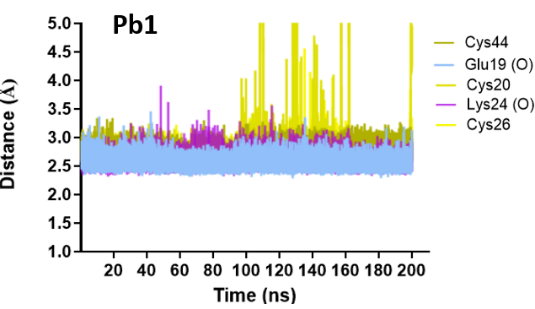** | **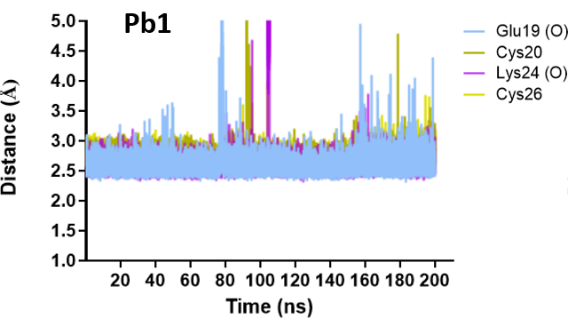** | **Pb1**  **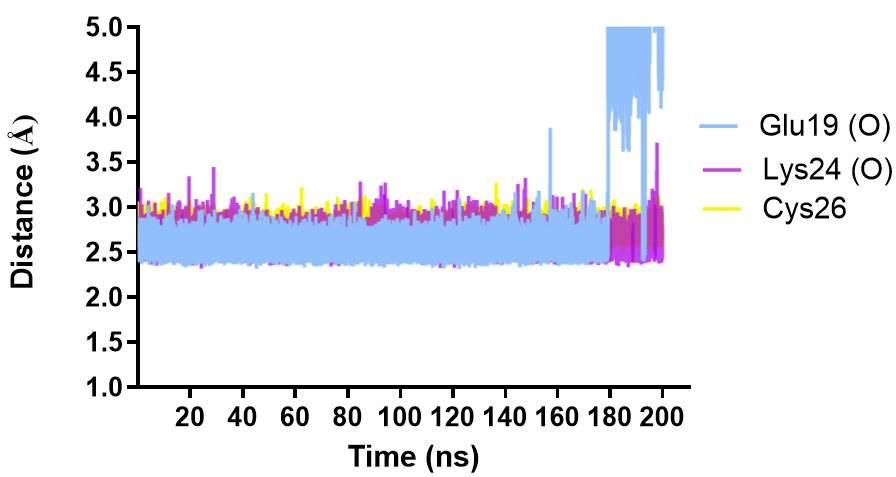** |
| 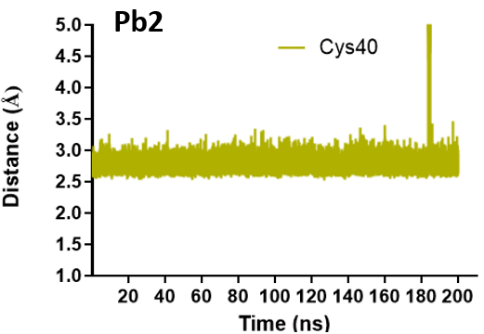 | 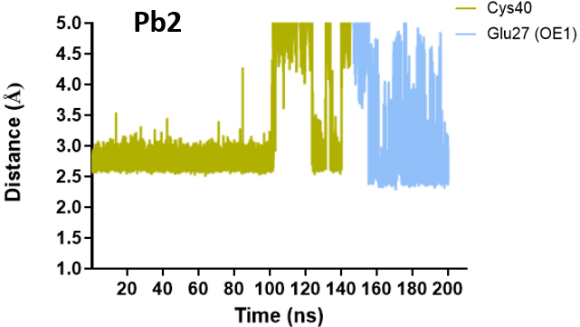 | **Pb2**  **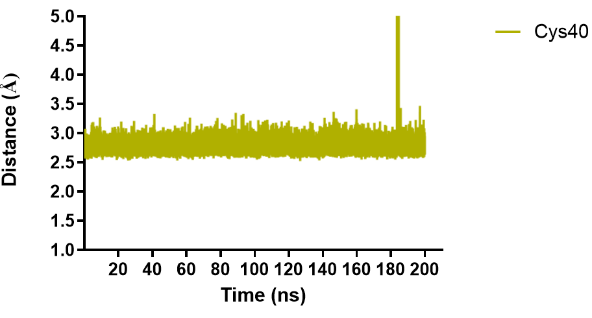** |
| 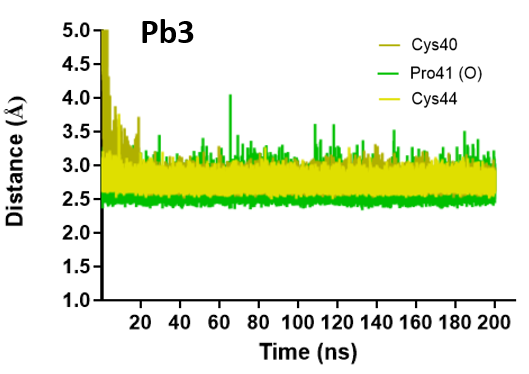 | **Pb3** | **Pb3**  **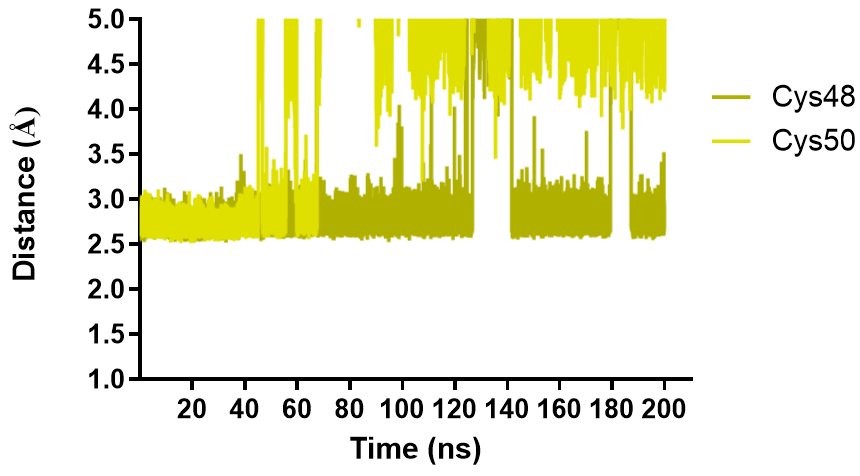** |
| 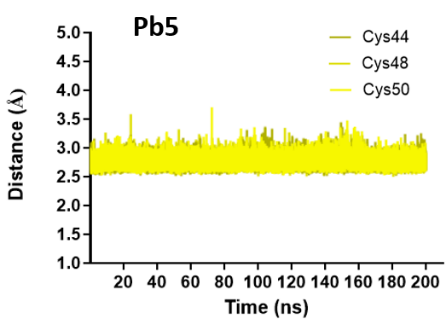 | **Pb5** | **Pb5**  **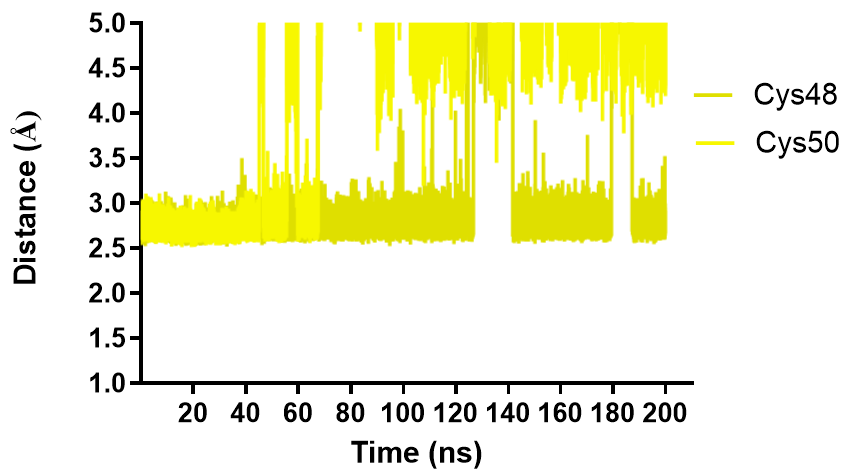** |
| 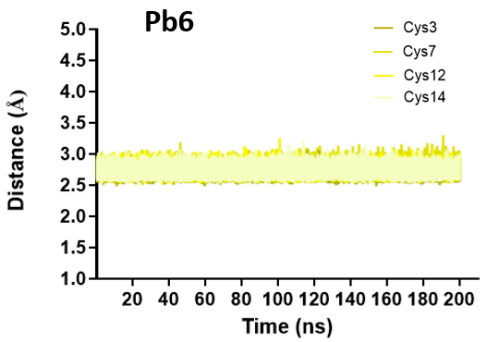 | 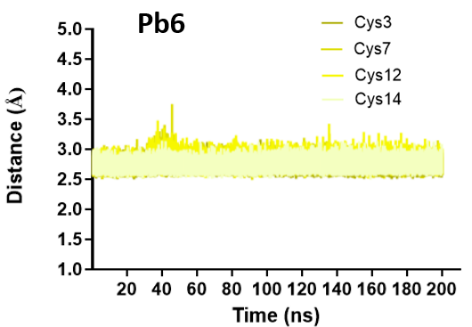 | **Pb6** |
|  |  | **Pb7** |
| **Figure S9.** Distances greater than 3 Å between residues and the Pb ion at each coordination site. Pb3 and Pb4 ions from Replica 2, and the Pb7 ion from Replica 3, did not coordinate with any residues within 3 Å. The Pb4 ion showed no coordination with any residues at this distance. | | |
